# Structural Basis for Properdin Oligomerization and Convertase Stimulation in the Human Complement System

**DOI:** 10.3389/fimmu.2019.02007

**Published:** 2019-08-22

**Authors:** Dennis V. Pedersen, Trine A. F. Gadeberg, Caroline Thomas, Yong Wang, Nicolas Joram, Rasmus K. Jensen, Sofia M. M. Mazarakis, Margot Revel, Carine El Sissy, Steen V. Petersen, Kresten Lindorff-Larsen, Steffen Thiel, Nick S. Laursen, Véronique Fremeaux-Bacchi, Gregers R. Andersen

**Affiliations:** ^1^Department of Molecular Biology and Genetics, Center for Structural Biology, Aarhus University, Aarhus, Denmark; ^2^Service d'Oncologie Pédiatrique, CHU Nantes, Hôpital Mère Enfant, Nantes, France; ^3^Department of Biology, Linderstrøm-Lang Centre for Protein Science, University of Copenhagen, Copenhagen, Denmark; ^4^Service de Réanimation Pédiatrique, CHU Nantes, Nantes, France; ^5^Centre de Recherche des Cordeliers, INSERM, Sorbonne Université, USPC, Université Paris Descartes, Université Paris Diderot, Paris, France; ^6^Service d'Immunologie Biologique, Assistance Publique – Hôpitaux de Paris, Hôpital Européen Georges Pompidou, Paris, France; ^7^Department of Biomedicine, Aarhus University, Aarhus, Denmark

**Keywords:** complement, convertase, properdin, complement component C3, crystal structure, regulation, factor B

## Abstract

Properdin (FP) is a positive regulator of the immune system stimulating the activity of the proteolytically active C3 convertase C3bBb in the alternative pathway of the complement system. Here we present two crystal structures of FP and two structures of convertase bound FP. A structural core formed by three thrombospondin repeats (TSRs) and a TB domain harbors the convertase binding site in FP that mainly interacts with C3b. Stabilization of the interaction between the C3b C-terminus and the MIDAS bound Mg^2+^ in the Bb protease by FP TSR5 is proposed to underlie FP convertase stabilization. Intermolecular contacts between FP and the convertase subunits suggested by the structure were confirmed by binding experiments. FP is shown to inhibit C3b degradation by FI due to a direct competition for a common binding site on C3b. FP oligomers are held together by two sets of intermolecular contacts, where the first is formed by the TB domain from one FP molecule and TSR4 from another. The second and largest interface is formed by TSR1 and TSR6 from the same two FP molecules. Flexibility at four hinges between thrombospondin repeats is suggested to enable the oligomeric, polydisperse, and extended architecture of FP. Our structures rationalize the effects of mutations associated with FP deficiencies and provide a structural basis for the analysis of FP function in convertases and its possible role in pattern recognition.

## Introduction

**Figure 1:**
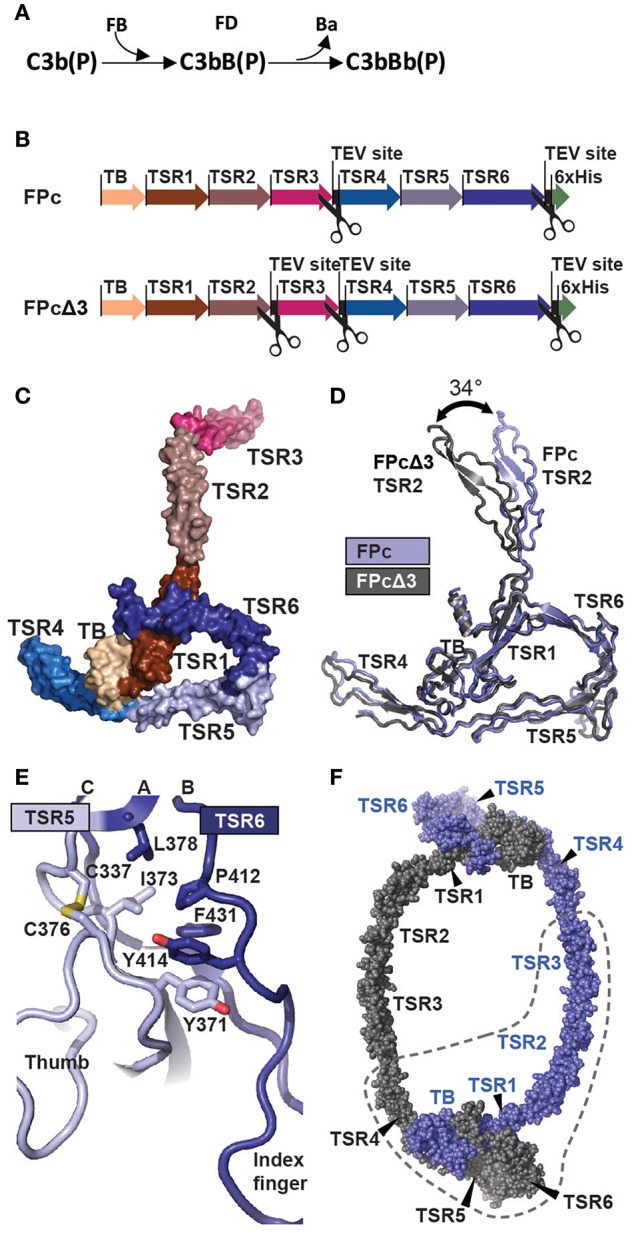
The function and structure of FP. **(A)** In the complement alternative pathway FP bound C3b can bind FB forming the C3bB(P) AP C3 proconvertases. The serine protease FD can then cleave zymogen FB resulting in the AP C3 convertase C3bBb(P). According to nomenclature, the letter “F” is skipped in complexes of complement proteins. **(B)** Cartoon of the FPc and FPcΔ3 constructs. The domains are indicated by a thick arrow in the color corresponding to the specific domain. **(C)** A surface representation of the FPc crystal structure with TB domain and TSR coloring as in panel B. **(D)** Overlay of the structures of FPc (blue) and FPcΔ3 (gray) shown in a cartoon representation demonstrating the flexibility of the TSR2 domain. **(E)** A cartoon representation of the interface in FP between TSR5 (light blue) and TSR6 (navy blue). Residues important for the interface are shown as sticks. **(F)** Theoretical model of a FP dimer with the monomers colored gray and blue, respectively. The dashed line outlines one FPc molecule for comparison with panel C.

The complement system is a tightly regulated proteolytic cascade, central to the innate immune system. It is involved in the detection, phagocytosis and killing of invading pathogens, as well as clearance of immune complexes. The complement system is furthermore involved in maintenance of host homeostasis (1) and is important during neural development (2). Activation of the cascade depends on recognition of pathogen-associated molecular patterns or danger-associated molecular patterns on activators such as pathogens and apoptotic host cells. Activation can occur through three pathways known as the classical (CP), the lectin (LP), or the alternative pathway (AP). The point of convergence is the cleavage of the 186 kDa complement component 3 (C3) generating the C3b fragment (178 kDa) which becomes covalently associated with the activator surface. C3b can then associate with the zymogen form of factor B (FB) forming the AP C3 proconvertase (Figure 1A). C3b bound FB is activated by the serine protease factor D (FD) forming the smaller fragment Ba and the larger fragment Bb (60 kDa), which remains non-covalent associated with C3b. The C3bBb complex is the AP C3 convertase, which catalyzes further C3 cleavage.

Several proteins within the complement system downregulate the activity of the AP convertase through decay accelerating activity (convertase dissociation) and cofactor activity, enabling factor I (FI) degradation of C3b into iC3b. In contrast, properdin (FP) is a direct positive regulator of the AP and stabilizes the convertase by associating with C3b in the convertase and proconvertase, reviewed (3). The function and importance of FP in host immunity is illustrated by the severity of disease in X-linked FP deficiencies giving rise to three different phenotypes (4). The hallmark of type I deficiency is a complete lack of FP, type II results in a low level of FP in plasma and type III is characterized by a dysfunctional FP present at a plasma concentration in the normal range (5). All three types of deficiencies are characterized by low serum AP activity and increased susceptibility to meningococcal infection and sepsis (6). FP deficiencies may also be related to autoimmune diseases such as systemic lupus erythematosus (7).

It has been predicted for 30 years that the majority of FP is organized in six thrombospondin repeats (TSRs) (8). The nascent FP monomer is secreted into the endoplasmic reticulum where it undergoes extensive posttranslational modification adding up to 15 C-linked, one N-linked and four O-linked glycans to the polypeptide chain (9, 10). FP oligomerizes prior to secretion, and in plasma it is found in a mass distribution with roughly 25% tetramers, 50% trimers and 25% dimers (11, 12). FP is produced predominantly by monocytes, T-cells and neutrophils and circulates in plasma at ~4–25 μg/ml (13). The capability of stimulating the alternative pathway increases strongly with oligomer size (11).

Numerous crystal and electron microscopy (EM) structures determined the last two decades have provided a comprehensive structural framework that now underlies the mechanistic understanding of the complement system and supports the development of therapeutic strategies aiming at complement modulation, reviewed in Morgan and Harris (14) and Schatz-Jakobsen (15). But, the oligomerization and polydispersity of FP complicate structural studies. Classic studies conducted with negative stain EM revealed that FP oligomers are extended structures organized in cyclic dimers, trimers, and tetramers in which doughnut shaped vertexes formed by two protomers are connected by thin flexible structures (16). Solution scattering of FP indicated a maximum extent of 25 and 30 nm for the FP dimer and trimer, respectively (17). Recently, a more detailed negative stain EM study using oligomeric FP confirmed the presence of cyclic oligomers. Furthermore, it was observed that each FP vertex contains one binding site for the AP C3 convertase and it was suggested that the FP vertex contacts both C3b and Bb (18).

Due to the polydispersity and flexibility of FP observed by EM, we recently engineered a recombinant oligomeric FP from which a functional monomer called FPc could be excised by specific proteolysis. This was accomplished by insertion of a cleavage site for the TEV protease between the third and fourth FP thrombospondin repeats (12). This location of the cleavage site was based on prior results demonstrating that FP with the third thrombospondin repeat deleted forms oligomers and stabilize the C3bBb convertase (19). The functional monomer FPc encompass residues 28–255 (the head fragment) from one monomer and residues 256–469 (the tail fragment) from a second monomer (Figure 1B). Using FPc, we showed by low resolution crystallography that FP mainly contacts C3b in the convertase C3bBb (12, 18) in accordance with the results presented in Alcorlo et al. (18). However, in the absence of an atomic model of FP, identification of FP and convertase residues in contact and the exact organization of the FP monomers and oligomers remained unsettled. Here we present the first atomic structures of FP allowing us to describe the contacts between two FP molecules responsible for oligomer formation and to propose an atomic model for the FP-C3bBb complex.

## Materials and Methods

### Protein Production

#### Properdin

TEV protease cleavable FP was expressed from a stably transfected HEK293F cell line generated as described in Pedersen et al. (20). WT FP was expressed by transient expression in HEK293F cells as described in Pedersen et al. (12). WT FP and FPc generated from TEV cleavable FP were both purified as described (12). His-tagged FPc and FPcΔ3 used for crystallization were expressed and purified from HEK293F cell supernatants as described (20). DNA encoding the FP TB-TSR3 head fragment (FPh) and TSR4-6 tail fragment (FPt) was synthesized (GenScript) with the endogenous FP signaling peptide and a 6His-tag at the C-terminus of FPt. FPh and FPt were then cloned into separate pCEP4 mammalian expression vectors using HindIII and BamHI restriction sites. Constructs encoding FP TB-TSR1 (FPhΔ2,3) and TB-TSR2 (FPhΔ3) were generated from FPh by insertion of a stop codon between TSR1 and TSR2 or TSR2 and TSR3, respectively, by site-directed mutagenesis. In the FPhΔ2,3 construct C132 was mutated to Ala to avoid a free cysteine. Co-transfection of FPh, FPhΔ3, or FPhΔ2,3 with FPt results in the expression of FPht, FPhtΔ3, and FPhtΔ2,3, respectively. FPht, FPhtΔ3 and FPhtΔ2,3 were expressed by transient transfection of HEK293F cells as described (12). FPht variants and FPhtΔ3 and FPhtΔ2,3 were purified from cell supernatants as described (20).

#### Native C3, C3b, biotin-C3b, hC3Nb1, FB, miniFH, FH, FI, and FD

Native C3 was purified from outdated plasma pools as described previously (21). The native C3 used for crystallization was obtained by a final size exclusion chromatography (SEC) purification step on a Superdex200 increase 24 mL (GE Healthcare) in 20 mM HEPES, 150 mM NaCl pH 7.5. C3b was generated by trypsination of native C3 and purified as described in Pedersen et al. (12). Biotinylated C3b was generated from C3 with 10 fold molar excess of Maleimide-PEG2-Biotin reagent being present during the cleavage of native C3. The generated C3b further was incubated with the Maleimide-PEG2-Biotin reagent for 6 h on ice after cleavage before being purified by cation exchange. hC3Nb1 was expressed and purified as described (21). A codon optimized version of the miniFH construct similar to that in Schmidt et al. (22) was synthesized (GenScript) with a C-terminal TEV-site followed by a 6His-tag and inserted into pcDNA3.1. MiniFH was expressed in HEK293F cells after PEI transfection and purified by affinity chromatography followed by anion-exchange on a 1 mL Source15Q column equilibrated in 20 mM Tris pH 8.8. The miniFH was eluted by a linear gradient from 50 to 400 mM NaCl. A final polishing SEC step was performed on a 24 mL Superdex200 increase equilibrated in 20 mM HEPES, 150 mM NaCl pH 7.5. FB variants K348A, L349A, K350A were generated by site directed mutagenesis using FB D279G, S699A as template. Recombinant FB D279G, S699A and FB D279G, S699A, K348A, L349A, K350A were expressed and purified from HEK293F cells as described (12). FD, FI, and FH were obtained from Complement Technology.

#### Selection and Production of hFPNb1

One llama (Lama glama) was immunized 4 times with FPc (400 μg) to generate a robust immune response. Blood was collected and shipped to Aarhus University where peripheral blood lymphocytes were isolated and used for total RNA extraction using the RNase Plus Mini Kit (Qiagen). cDNA encoding VHHs was generated using the SuperScript III First-Strand Kit (Invitrogen) with random hexamer primers and amplified by PCR using primers specific for the VHH genes. The PCR product was subsequently cloned into a pCANTAB phagemid vector for phage display. The M13 phage-display Nb library was generated using the trypsin cleavable M13KO7^trp^ helper phage. Selection was performed with 1 μg of FPc coated in a microtiter well. Competitive elution was performed by adding 100 μL of 1 mg/mL polyclonal goat anti-FP Ab (Complement Technology) in PBS. After 30 min, the well was washed thrice with PBS and the remaining phages were eluted using trypsin (10 μg/mL in PBS) for 30 min at 37°C. The eluted phages were then added to 0.9 mL mid-log phase *E. coli* TG1 culture (OD_600_ 0.4–0.5) for infection at 37°C for 1 h. Bacteria were grown overnight at 30°C and harvested for phage amplification and a second round of selection performed with 0.1 μg of FPc. After two rounds of selection, single colonies were transferred to a 96-well plate where Nb-pIII was expressed at 30°C overnight using 1 mM IPTG. The 96-well plate was subsequently centrifuged and the supernatant was analyzed by ELISA. MaxiSorp microtiter wells (Nunc) were coated with 1 μg FPc overnight and blocked for 1 h with 2% w/v BSA/PBS. 50 μl supernatant were then transferred to each well and incubated for 1 h. The wells were subsequently washed with PBS-T and 50 μl HRP-anti-E-tag mAb diluted 1:5,000 in 2% w/v BSA/PBS was added. After 1 h incubation, the wells were washed with PBS and 100 μL TMB (Thermo Scientific) was added to each well. The reaction was stopped with 50 μL 1 M H_2_SO_4_ and the plate was read at OD_450_ and OD_650_. Positive clones were subcloned into the pET-22b(+) expression vector for bacterial expression. hFPNb1 was subsequently expressed and purified as described in Jensen et al. (21).

#### Complement Deposition

To assay the effect of hFPNb1 on AP activity microtiter wells were coated with zymosan (2 μg/mL in 50 mM sodium carbonate buffer, pH 9.6) and washed thrice with TBS containing 0.1% Tween 20 (TBS-T) before blocking wells with 1 mg/mL HSA in TBS-T. Fifty μL sample containing 0.007–714 nM hFPNb1 in NHS diluted 1:10 in VBS/EGTA/Mg^2+^ buffer (5 mM barbital, pH 7.4, 145 mM NaCl, 10 mM EGTA, 5 mM MgCl_2_) was added to each well and the plates were incubated at 37°C for 1 h. A control experiment was performed with NHS diluted in VBS/EDTA (VBS supplemented with 10 mM EDTA). After incubation, wells were washed thrice with TBS-T and 50 μL of 0.75 μg/mL biotin-anti-hC3c (Dako) in TBS-T was added to each well and plates incubated overnight at 4°C. The following day, wells were washed thrice with TBS-T before 50 μL of 1 μg/mL Europium-labeled streptavidin (Perkin-Elmer) in TBS-T supplemented with 25 μM EDTA was added to each well and incubated for 1 h at room temperature. The wells were then washed with TBS-T, and 50 μL DELFIA enhancement solution (Perkin-Elmer) was added to each well. The plates were vortexed vigorously and incubated for 2 min before time-resolved fluorescence was measured at 615 nm with a VICTOR5 plate reader (PerkinElmer).

#### X-ray Structure Determination

Crystallization of FPc and FPcΔ3 and the data quality have been described elsewhere (20) and details for C3bBbSCIN-FPc crystallization and data collection were also described (12). A sulfur-SAD data set was obtained from two crystals of FPcΔ3 at the Petra III P13 beamline with a helium path inserted between the crystal and the detector. Crystals of the C3bBbSCIN-FPhtΔ3-hFPNb1 complex were obtained by vapor diffusion against a reservoir solution containing 100 mM NaCl, 5% (w/v) PEG4000, 10 mM MgCl_2_, 100 mM Sodium Cacodylate trihydrate pH 5.8. Crystals of hFPNb1 were obtained by vapor diffusion of 450 nL drops mixed in a 1:2 ratio with a reservoir containing 13.1% (v/v) MPD, 13.1% PEG1000 (w/v) 13.1% (w/v) PEG3350, 100 mM Tris Bicine pH 8.5, 33 mM CaCl_2_, 33 mM MgCl_2_, 4% (v/v) formamide, 114 mM FOS-Choline 8. Crystals of C3bBbSCIN-FPhtΔ3-hFPNb1 were cryoprotected by soaking in reservoir solution supplemented with 25% glycerol whereas hFPNb1 crystals were cryoprotected directly in reservoir solution prior to data collection at Petra III. Native C3 at a final concentration of 8 mg/mL was added 1.5 fold molar excess of hC3Nb1 and crystallized by vapor diffusion of 200 nL drops mixed in a 1:1 ratio with a reservoir containing 100 mM HEPES pH 7.2, 1.75% (w/v) PEG8000. The crystals were cryoprotected in reservoir solution supplemented with 30% glycerol prior to data collection at Petra III. All diffraction data (Supplementary Table 2) were processed with XDS (23). The FPcΔ3 structure was determined by molecular replacement. Electron densities for the C3bBbSCIN-FPc complex (12) and the C3bBbSCIN-FPhtΔ3-hFPNb1 complex (this study) calculated without contribution from FP models were improved by multi crystal averaging with phenix.multi_crystal_average (24). The resulting density was used for constructing a poly-alanine model for TSR1 and TSR4-6 based on known structures of TSRs, while multiple model secondary structure fragments were placed into density for the TB domain. This polyalanine model was used to isolate a density based search model roughly encompassing the TSR1, TSR5, TSR6, and the TB domain with phenix.cut_out_density (24). Molecular replacements solutions for the FPcΔ3 native data (20) and the FPcΔ3 sulfur SAD data (Supplementary Table 2) were identified with phenix.phaser (25) and subjected to density modification with 81% solvent. After improvement with phenix.multi_crystal_average over the two different FPcΔ3 crystals, the resulting phases were used to identify sulfur sites in the anomalous data. Although a pure experimental map calculated with phenix.autosol by SAD phasing and density modification starting from these sites revealed the outline of FPcΔ3, the inclusion of these phases did not improve the electron density compared to the MR based multi crystal averaging map already obtained. However, most of the disulfide bridges in the TB and TSR domains could be mapped to the sulfur sites identified in the anomalous data. An iterative process involving rebuilding in Coot (26) and refinement with phenix.refine (27) provided an initial model containing all FPcΔ3 domains. The TB domain fold and disulfide bridge arrangement were confirmed through a DALI search (28). This FPcΔ3 model was then used for molecular replacement into the 2.8 Å FPc data, and a complete model for FPc was build and refined in an iterative manner. The structure of hFPNb1 was determined by molecular replacement followed by rebuilding and refinement. The final model of FPc was placed manually into the density modified maps of the C3bBbSCIN-FPc and C3bBbSCIN-FPhtΔ3-hFPNb1 complexes. In the latter complex, clear omit density allowed the docking of the refined hFPNb1 structure. The resulting models of the two complexes were refined with phenix.refine using rigid body and TLS refinement, positional refinement under NCS restraints, and grouped B-factor refinement. Side chains at the FP-convertase interface were remodeled if the associated main chain was altered or clashes occurred, but to avoid bias side chains were not fitted in detail to point toward putative interaction partners. The structure of the C3-hC3Nb1 complex was determined using the coordinates of the C3 structure (PDB entry 2A73) with the C345c domain removed for molecular replacement in phenix.phaser. The coordinates of hC3Nb1 (RCSB entry 6EHG) and the C345c domain (RCSB entry 2A73) were manually placed in Coot. The model was then refined against the reflections with rigid body, TLS and grouped B-factor refinement in phenix.refine. In an iterative manner the structure was subjected to cycles of manual rebuilding in coot followed by positional, grouped B-factor and TLS refinement in phenix.refine. Prior to final refinement the structure was fitted to the electron density map using iMDFF as described in Croll and Andersen (29). Diffraction data and atomic models are available at the protein data bank as entries 6RUS, 6SEJ, 6RUR, 6RU3, 6RUV, 6RU5, 6RV6 as specified in Supplementary Tables 1, 2.

#### Mass Spectrometry

Purified FPcΔ3 in 50 mM ammonium bicarbonate, 5 mM iodoacetamide was heated to 60°C for 10 min in the presence of 0.1% (v/v) of the RapiGest (Waters) denaturant, allowed to cool and subsequently digested at 37°C for 16 h by the addition of porcine trypsin (1:20). The digest was acidified by the addition of 0.5% trifluoroacetic acid and incubated at 37°C for 1 h to allow RapiGest degradation. The generated peptides were separated by UPLC reverse-phase chromatography on a BEH300 C18 column (2.1 mm × 15 cm; 1.7 μm) operated by an Aquity UPLC system (Waters). The column was run at 300 μL/min using a 1% B/min linear gradient of solvent B (90% acetonitrile, 0.08% (v/v) trifluoroacetic (TFA) in solvent A (0.1% (v/v) TFA). Fractions were collected manually and analyzed by MALDI mass spectrometry on a Bruker Autoflex III instrument operated in linear mode and calibrated in the mass range of 5,000 Da to 17,500 Da using Protein calibration standard I (Bruker Daltronics). The analysis was performed in linear mode to enable the detection of disulfide-linked peptides.

#### FH-Assisted FI Cleavage Assay

High capacity streptavidin agarose beads (Thermo Scientific) were saturated with biotinylated C3b, yielding ~8 μg of C3b per μL of settled streptavidin beads. The streptavidin beads were added 4% (w/w) FH and 2-fold molar excess of oligomeric FP as compared to C3b. The beads were incubated for 30 min on ice before 1% (w/w) of FI compared to C3b was added. The reactions were incubated at 37°C and samples were taken after 15, 30, 60, and 120 min, and analyzed by SDS-PAGE. The ratio of the C3b α' chain and streptavidin was quantified from three independent experiments using ImageJ (30). The FP used in this assay was ~50% dimer and 50% trimer as judged by SEC analysis performed on a 24 mL Superdex200 increase.

#### Bio-Layer Interferometry Assays

Bio-layer interferometry experiments were performed on an Octet Red96 (ForteBio) at 30°C and shaking at 1,000 RPM. For the FP/FH competition assay, miniFH was immobilized on amine reactive sensors (AR2G, ForteBio). The sensors were equilibrated in H_2_O for 5 min before being activated in a mixture of 20 mM 1-ethyl-3-(3-dimethylaminopropyl)-carbodiimide and 10 mM N-hydroxysuccinimide for 5 min. MiniFH was then loaded at 20 μg/mL in 10 mM sodium acetate, 100 mM NaCl pH 5.0 for 10 min before the sensors were quenched with 1 M ethanolamine for 5 min. The sensors were equilibrated in the assay buffer (PBS supplemented with 1 mg/mL BSA and 0.05% Tween 20) for 5 min. The association of either FP alone (94 nM), C3b (280 nM), or a mixture of FP and C3b (94 and 280 nM, respectively) were assessed for 60 s followed by a 60 s dissociation step in assay buffer. The assay was repeated twice after regeneration of the sensors using PBS supplemented with 4 M of NaCl. The FP used for the assay was ~90% dimer and 10% trimer as judged by SEC analysis performed on a 24 mL Superdex200 increase. For kinetic analysis hFPNb1 mutants were immobilized on Anti-Penta-HIS sensors (HIS1K, ForteBio). The sensors were equilibrated in assay buffer (PBS supplemented with 1 mg/mL BSA and 0.05% Tween 20) for 5 min before the sensors were conditioned by running a regeneration cycle consisting of three sub-cycles of 5 s in 100 mM glycine pH 2.5 followed by 5 s in assay buffer. The sensors were subsequently equilibrated for 60 s in assay buffer before being loaded with 50 μg/mL of hFPNb1 variant in assay buffer for 120 s. The sensors were then equilibrated in assay buffer for 60 s followed by an association phase of 120 s with FPc at 189, 94.5, 47.3, 23.6, 11.8, or 5.9 nM. This was followed by a 120 s dissociation step in assay buffer. The sensors were then subjected to a new round of regeneration before being used for a new round of kinetics. The experiment was first performed with hFPNb1, followed by hFPNb1 I103A, hFPNb1 I103A, L104A, and hFPNb1 I103A, L104A, V105A, respectively. The kinetic constants were determined using non-linear regression in GraphPad Prism. A 1:1 Langmuir binding model was applied where the association is modeled as R(t) = R_eq_(1-exp(-k_obs_·t), k_obs_ = k_on_·[FPc]+k_off_, R_eq_ = R_max_([FPc]/([FPc]+K_D_), K_D_ = k_off_/k_on_; and the dissociation is modeled as a first order exponential decay, R(t) = R(120)·exp(-k_off_(t-120s). For the BLI assay assessing the ability of FPht variants to bind C3b, biotinylated C3b was immobilized on streptavidin sensors (SA, ForteBio). The sensors were equilibrated in assay buffer (20 mM MES pH 6.0, 100 mM NaCl, 0.05% Tween, 1 mg/mL BSA) for 5 min. The sensor were then loaded with biotinylated C3b at 16 μg/mL for 10 min, before being equilibrated in assay buffer for 240 s. The association of 100 μg/mL FPht WT or mutants (R329A, R330A, Q343R, R351A, R353A, R359A, Q364A, Q365A, Q364A/Q365A, Y414D) were then assessed in a 120 s association step followed by a 120 s dissociation step in assay buffer. Two independent but identical experiments were performed. The R_eq_ was determined as the average response from 80 to 120 s. The experiment quantifying the binding affinity of FPht WT, FPht R353A, FPht Q343R, and FPht Y414D was performed identically but with the association and dissociation steps reduced to 60 s and using FPht variants used at 100, 50, 25, 12.5, 6.25, 3.13, and 1.56 μg/mL. Due to the low level of binding of the FPht mutants the K_D_ was determined using steady state kinetics, by non-linear fitting in GraphPad Prism. A 1:1 binding model was applied where the R_eq_ was modeled as R_eq_ = R_max_C/(C+K_D_). The R_eq_ was determined as the average response from 40 to 60 s during the association phase. The K_D_ determined for FPht WT was very similar using steady state analysis (320 nM) and kinetic analysis using the integrated rate equations (350 nM).

For the assay analyzing the binding of FPht to C3b—FB (D279G, S699A, K348A, L349A, K350A), hFPNb1 was immobilized on amine reactive sensors (AR2G, ForteBio). The sensors were equilibrated in H_2_O for 5 min before being activated in a mixture of 20 mM 1-ethyl-3-(3-dimethylaminopropyl)-carbodiimide and 10 mM N-hydroxysuccinimide for 5 min. hFPNb1 was then loaded at 20 μg/mL in 10 mM sodium acetate pH 5.0 for 10 min before the sensors were quenched with 1 M ethanolamine for 5 min. The sensors were equilibrated in the assay buffer (20 mM MES pH 6.0, 100 mM NaCl, 0.05 % Tween, 1 mg/mL BSA, 5 mM MgCl_2_) for 5 min, before FPht (20 μg/mL) was loaded. The association of 50 μg/mL C3b, 50 μg/mL FB (D279G, S699A), 50 μg/mL FB (D279G, S699A, K348A, L349A, K350A), 50 μg/mL C3b + 50 μg/mL FB (D279G, S699A), or 50 μg/mL C3b + 50 μg/mL FB (D279G, S699A, K348A, L349A, K350A) was assessed for 120 s, followed by 120 s of dissociation in assay buffer. The signal for FB (D279G, S699A, K348A, L349A, K350A) or FB (D279G, S699A) were subtracted for their respective experiment in complex with C3b. A control experiment was performed where the FB (D279G, S699A) and FB (D279G, S699A, K348A, L349A, K350A) binding to a C3b coated streptavidin sensor was tested (SA, ForteBio). The sensors were equilibrated in assay buffer (20 mM MES pH 6.0, 100 mM NaCl, 0.05% Tween, 1 mg/mL BSA, 5 mM MgCl_2_) for 5 min. The sensor were then loaded with biotinylated C3b at 16 μg/mL for 10 min, before being equilibrated in assay buffer for 240 s. The association of 40 μg/mL FB (D279G, S699A) or 40 μg/mL FB (D279G, S699A, K348A, L349A, K350A) where then assessed in a 120 s association step followed by a 120 s dissociation step in assay buffer.

#### Analysis of FP Folding and Expression

HEK293F cells were co-transfected with either FPh L47A + FPt, FPh L58A + FPt, FPh L47A, L58A + FPt, FPh + FPt L275A, FPh L47A, L58A + FPt L275A, FPh + FPt L456A, FPh L47A, L58A + FPt L456A, FPh C32Y + FPt, FPt, FPh, FPh + FPt, or WT FP in a total of 50 mL per transfection. The cells were harvested after 4 days by centrifugation at 200 × g, and the cell pellet were frozen. The cell supernatants were centrifuged at 6,000 g and subsequently filtered through a 0.22 μm filter. The cell pellets were thawed and resuspended in 30 mL of 25 mM Tris, 150 mM NaCl pH 7.6, 1% (w/v) nonyl phenoxypolyethoxylethanol, 1% sodium deoxycholate, 0.1% (w/v) SDS, 1 mM phenylmethylsulfonyl fluoride and 5 mM EDTA. The cells were lyzed by sonication, the cell debris was removed by centrifugation at 14,000 g for 10 min and the supernatant was filtered through a 0.22 μm filter. Total protein content was determined by a Bradford assay (BioRad) using BSA as a standard to quantitate protein content of samples. This was used to normalize the sample volume loaded on a 4–20% SDS-PAGE (GenScript) of both the cell pellet and cell supernatant. PVDF membranes were activated in 96% ethanol for 5 min and the protein was blotted from the SDS-PAGE gel onto a membrane in 25 mM Tris, 192 mM glycine, 20% (v/v) ethanol, pH 8.3, for 1 h at 100 V. The blots were blocked for 1 h in PBS-T supplemented with 3% BSA and subsequently washed for 5 min in PBS-T. The blots were incubated O/N in PBS-T added primary antibody at 4°C. The blot containing the cell pellet was added rabbit anti-GAPDH antibody (Santa Cruiz Biotechnology) diluted 1:2,000 and the blot containing the cell supernatant was added mouse antiHis mAb (GenScript) diluted 1:4,000. The blots were washed thrice for 5 min in PBS-T and subsequently incubated for 1 h at room-temperature in PBS-T added secondary antibody. For the blot containing the cell pellet goat anti-rabbit HRP-conjugated antibody (Sigma-Aldrich) diluted 1:20,000 was added and for the blot containing the cell supernatant anti-mouse HRP-conjugated antibody (Jackson ImmunoResearch) diluted 1:15,000 was added. The blots were then washed thrice in PBS-T before being added SuperSignal West dura extended duration substrate (Thermo Scientific) for 5 min. The blots was developed on X-ray film.

#### Analysis of FP E244 Mutations

FP mutants E244K, E244A, E244R, and E244Q were generated by site directed mutagenesis using the QuickChange Lightning kit (Agilent technologies) and the pCEP4:FP construct described in Pedersen et al. (12) as template. WT FP and FP mutants were transfected into 100 mL HEK293F cells as described (12). The cells were harvested and the supernatants were filtered using 0.22 μm filters. The pH was adjusted by addition of 20 mM Tris pH 8.0. Fifty μg of hFPNb1 were added to each supernatant and incubated for 2 h at room temperature. Twenty-five μL of Ni-NTA beads were then added and the samples were incubated O/N at 4°C. The beads were transferred to 1 mL spin columns (Biorad) and the beads were washed thrice with 500 μL of 100 mM HEPES, 500 mM NaCl, 10 mM imidazole pH 7.5. The bound hFPNb1 complexes were subsequently eluted in 300 μL of 100 mM HEPES, 500 mM NaCl, 400 mM imidazole pH 7.5. Eluates were retrieved by centrifugation at 70 g for 30 s and subsequently reapplied to the column before another centrifugation step was performed. The eluted FP variants as well as plasma purified FP (Complement Tech) were applied to a 24 mL Superdex200 increase equilibrated in 20 mM HEPES, 150 mM NaCl, pH 7.5. Fractions of 250 μL were collected throughout the run and every second fraction from 8 mL until 13 mL was analyzed by western blotting. The samples were loaded on a 4–20% SDS-PAGE (GenScript) and were subsequently transferred to a nitrocellulose membrane. The membrane was blocked O/N at 4°C in MPBS (PBS supplemented with 2% (w/v) skimmed milk powder). The membrane was then added goat anti-human FP antibody (Complement Technology) diluted 1:2,000 in MPBS for 3 h at room temperature. The membrane was washed in PBS-T followed by two washes in PBS for 5 min each. Rabbit anti-goat HRP-conjugated antibody (Sigma-Aldrich) diluted 1:2,000 in MPBS was added and incubated for 2 h at room temperature. The membrane were then washed thrice in PBS-T before being added SuperSignal West dura extended duration substrate (Thermo Scientific) for 5 min. The blots was developed on X-ray film.

#### Patient Data

A patient with a FP deficiency was identified in the Complement Diagnostic Laboratory at the HEGP hospital in Paris (with informed consent from the patient's parents for the genetic analyses) using direct sequencing. Complement analysis was performed in the plasma of the patient and his relatives. A CH50 test to evaluate the activity of the classical complement pathway was performed on sheep IgG-opsonized erythrocytes. An AP50 test to evaluate the alternative pathway activity was carried out on rabbit erythrocytes. Plasma C3 and C4 were quantified using nephelometry (Dade Behring, Deerfield, IL, USA). The levels of FP were evaluated by ELISA. All analyses were performed according to the routine diagnostic procedures of the Complement Diagnostic Laboratory at the HEGP hospital in Paris.

#### Molecular Dynamics Simulations

As starting point for our simulations, crystal structures of C3 (PDB entry 2A73) and C3b (PDB entry 5FO79) were placed into a rhombic dodecahedron box, solvated with TIP3P water molecules containing Na^+^ and Cl^−^ ions at 100 mM, resulting in ~371,000 atoms in the C3 model, and ~449,000 atoms in the C3b model. The disulfide bonds present in the crystal structures were maintained. The CHARMM36m force field (31) was used for both protein and ions. Simulations were performed in the NPT ensemble (298K, 1 bar) using the V-rescale thermostat (32) with a 1 ps coupling constant, and the Parrinello-Rahman barostat with a 2 ps time coupling constant. A cut-off of 1.0 nm for both the van der Waals interactions and the short-range electrostatic interactions, with long-range electrostatics treated using PME. Neighbor searching was performed every 10 steps. We employed the hydrogen mass repartitioning technique (33) with a single LINCS iteration (expansion order 6) (34) and constrained the bonds involving hydrogen atoms using the LINCS algorithm, allowing simulations to be performed with an integration time step of 5 fs. All MD simulations were performed using Gromacs 2016.3 (35). One microsecond MD simulation was performed for both C3 and C3b. The root mean square fluctuation (RMSF) was used to quantify the flexibility of the proteins. To measure the dynamics of the whole C345c domain, the RMSF profiles were calculated from the trajectories aligned on the C3/C3b β-chain. To measure the intra-domain dynamics of the C345c domain, the RMSF profiles were calculated from the trajectories aligned on the central β-sheet of the C345c domain (residue 1544–1610). For a better visualization of the residue fluctuations of C345c domain, the residue RMSF values were mapped onto the corresponding crystal structures of the C345c domain in C3 and C3b.

## Results

### Structure Determination and Description of FP

To elucidate the detailed structural organization of FP, we determined the crystal structures of the functional properdin monomer FPc earlier described by us (12) at 2.8 Å. We also determined the structure of the FPcΔ3 protein at 3.5 Å of resolution where the third thrombospondin repeat (TSR3) is removed by proteolysis of TEV sites inserted at the TSR2-TSR3 and the TSR3-4 junctions in FP (20). The quality of the diffraction data was previously reported in Pedersen et al. (20). The two structures agree in all aspects including post-translational modifications, disulfide bridge pattern and quaternary structure (Figures 1B–D and Supplementary Tables 1, 2). The deduced disulfide bridge pattern and a homology search conducted with a preliminary model of FPcΔ3 showed that residues 28–76 folds into a TGFβ binding (TB) domain (Supplementary Figures 1A–D) and not a truncated thrombospondin repeat as earlier proposed (17). The six thrombospondin repeats located after the TB domain adopt the expected fold with three strands A, B, and C (Supplementary Figure 2A). Whereas, strands B and C at the C-terminal end of each TSR form an antiparallel two-stranded β-sheet, the A strand containing the WxxWxxWxxCxx(S/T)C motif is rippled due to the presence of two residues between each tryptophan. These tryptophan side chains are stacking with interdigitating arginine side chains from strands B and C as observed for other TSRs (36). Two surfaces in each TSR can be defined; a convex surface containing the Trp-Arg stack and a weakly concave surface opposite to the Trp-Arg surface.

FPc has an overall shape of an elongated closed triangle formed by the TB domain and TSR1 from one FP monomer and TSR4, TSR5, and TSR6 from the second monomer (Figures 1C,D). From this triangle the N-terminal end of TSR4 extends as a short arm whereas TSR2 and TSR3 form a long extended arm. In the FPc structure, Pro255 at the C-terminal end of TSR3 and Val256 at the N-terminal end of TSR4 are separated by 136 Å reflecting that they come from two different FP monomers. Compared to TSR1-4, TSR5-6 are unusual. In TSR5, instead of having three residues between the two cysteines in the WxxWxxWxxCxx(S/T)C motif and an O-linked fucose-glucose disaccharide as in TSR1-4 (see below), an ordered non-glycosylated loop (called the “thumb” below) is formed by residues 328–336 that protrude at the concave face of TSR5 at its C-terminal end (Figure 1E). TSR6 is even more unique due to 31 residues inserted between the Cys407-Cys439 disulfide bridge connecting the B- and C-strands. The TSR6 insert folds back over TSR5 and extends with a hairpin like loop (termed the “index finger”) together with the TSR5 thumb loop at the concave face of TSR5 (Figure 1E). As compared to the other neighboring TSR-TSR interfaces in FP, the TSR5-6 domain interface is unusually large (977 Å^2^). It is centered on a hydrophobic core formed by highly conserved residues from both repeats (Figure 1E). The numerous TSR5-TSR6 interactions support a rigid orientation between the two TSRs which is strongly bend compared to other pairs of neighboring TSRs in FP.

The FPc and FPcΔ3 structures reveal hinges at the TSR1-TSR2 and TSR4-TSR5 junctions. At the TSR1-TSR2 interface a disulfide bridge is formed between Cys132-Cys170 located in TSR1 and TSR2, respectively. Anyway, this disulfide does not prevent flexibility at the TSR1-TSR2 junction as the rotation of TSR2 relative to the TB-TSR1-5-6 triangle differ by 34° between the two structures (Figure 1D and Supplementary Figure 1E). In contrast, for TSR4 we only observed 6° difference in the orientation relative to the triangle, but weak electron density for the N-terminal end of TSR4 as compared to its C-terminal end suggests that TSR4 may have some internal flexibility also, possibly due to the presence of only two tryptophans in its WxxWxxWxxCxx(S/T)C motif (Supplementary Figure 2A). Since we do not observe TSR3 in the C3bBbSCIN-FPc complex (see below), we suggest that a hinge is located at the TSR2-TSR3 junction as well. The compact shape of the FP monomer associated with the E244K mutation (12) furthermore requires a hinge at the TSR3-TSR4 junction. This suggest the presence of the following four hinges between TSRs in FP: TSR1-2, TSR2-3, TSR3-4, and TSR4-5.

A simple model of the FP dimer in concordance with classic EM pictures of FP oligomers (16, 18) can now be constructed starting from the FPc structure. First, the TSR3 is rotated to obtain a less bend orientation relative to TSR2. Then the TSR3 C-terminus is placed close to the N-terminus of TSR4 in a second FPc molecule while the TB-TSR1-TSR5-TSR6 triangle remains unchanged. The resulting idealized dimer model presented in Figure 1F has 2-fold symmetry, but FP oligomers may well be dynamic and non-symmetric. It is furthermore conceivable that by combinations of rotations at the four TSR-TSR hinges, one may model structures resembling the trimers and tetramers also known from EM micrographs (16). The dimer model displayed in Figure 1F has a maximum extent of 23 nm which is in reasonable agreement with the 25 nm including a hydration layer estimated by small angle X-ray scattering. A slightly larger dimer model may also be obtained by further rotation of TSR4 relative to TSR5. In summary, our crystal structures reveal a stable core formed by the TB domain and three TSRs, whereas flexibility at four TSR-TSR junctions allows for the formation of extended FP oligomers where the structural cores are separated by thrombospondin repeats 2, 3, and 4.

### The Post-translational Modifications of FP

Whereas, the disulfide bridge pattern in six thrombospondin repeats were those expected, the three cysteine bridges 32–56, 43–73, and 57–75 in the N-terminal TB domain differed from those annotated in UNIPROT. We therefore confirmed the disulfide bridge pattern by two orthogonal techniques. First, an anomalous Fourier map calculated from data collected at λ = 2.48 Å (Supplementary Figure 1B) confirmed the three disulfide bridges. Second, we also isolated the TB domain from the TB-TSR1-TSR2 fragment of FPcΔ3 by reverse phase chromatography and performed mass spectrometry analysis. This revealed ions representing disulfide-linked peptides confirming the observed disulfide arrangement (Supplementary Figure 1F). One peptide obtained by trypsin digestion contained an ion of m/z 5112.5, which corresponds to a complex composed of the 4 disulfide-linked tryptic peptides spanning the N-terminal region of FP (m/z_calc_ 5112.8) (Supplementary Figure 1F). The TB domain disulfide bridge pattern was furthermore validated by the absence of masses representing peptides connected by Cys32-Cys72/75 (Supplementary Figure 1F, green and yellow; m/z_calc_ 2620.9) and Cys43-Cys56/57 (Supplementary Figure 1F, red and blue; m/z_calc_ 2492.9). In summary, comprehensive structural data and mass spectrometry analysis demonstrated for the first time that FP residues 28–76 fold into a TB domain.

FP contains a wealth of posttranslational modifications with sugars also shared by other thrombospondin repeat containing proteins. Up to three tryptophans in the WxxWxxWxxCxx(S/T)C motif in each TSR domain may be modified with a mannosylation through a covalent bond between the tryptophan indole CD1 atom and the C1 atom of the mannose. For both FPc and FPcΔ3 the electron densities are consistent with the 14 mannosylations annotated in UNIPROT, but in addition we observed significant mannosylation at Trp202 confirming a recent MS study (9). As an example, the electron density for the three mannosylations in TSR2 is displayed in Supplementary Figure 3A. The electron density is not equally strong for all mannosylations and it cannot be excluded that some of these have only partial occupancy. We modeled the Trp-bound α-mannosyl residues in the ^1^C_4_ chair conformation as it consistently fitted the density better than the default favored ^4^C_1_ pyranose conformation. In addition, NMR measurements on a model mannose-indole compound support the ^1^C_4_ chair conformation for the α-mannosyl linkage (37). We observe a preferred orientation of the pyranose ring where the 2-OH interacts with the tryptophan main chain amide (Supplementary Figure 3B), and the endocyclic pyranose oxygen and the 6-OH in many instances engage in hydrogen bonding with an arginine side chain stacking with the tryptophan.

In addition to tryptophan mannosylations, we also observe clear density for four O-linked fucosyl-glucosyl disaccharides linked to the threonine/serine side chain located at C-terminal end of the concave face in TSR1-4. In the TSR1 domain the O-linked glycosylation on Thr92 points toward TSR2 and shields the Cys93-Cys133 disulfide from solvent (Supplementary Figure 1E). In TSR2, Thr151 is modified and also in this case the disaccharide shields the equivalent Cys152-Cys190 disulfide but it also engages in a hydrogen bond with His193 in TSR3. In TSR3, the disaccharide linked to Ser208 likewise shields the Cys209-Cys254 disulfide. In TSR4, Thr272 carries the disaccharide that again shields the disulfide bridge Cys273-Cys312 but in addition forms a hydrogen bond with Asn59 in the TB domain. In summary, all the expected C-linked and O-linked posttranslational modifications are observed and well-ordered in our structures. The O-linked glycans appear to have a common structural function in protecting a nearby conserved disulfide bridge.

### Structure of the FP Convertase Complex and the Mechanism of AP Stimulation

**Figure 2:**
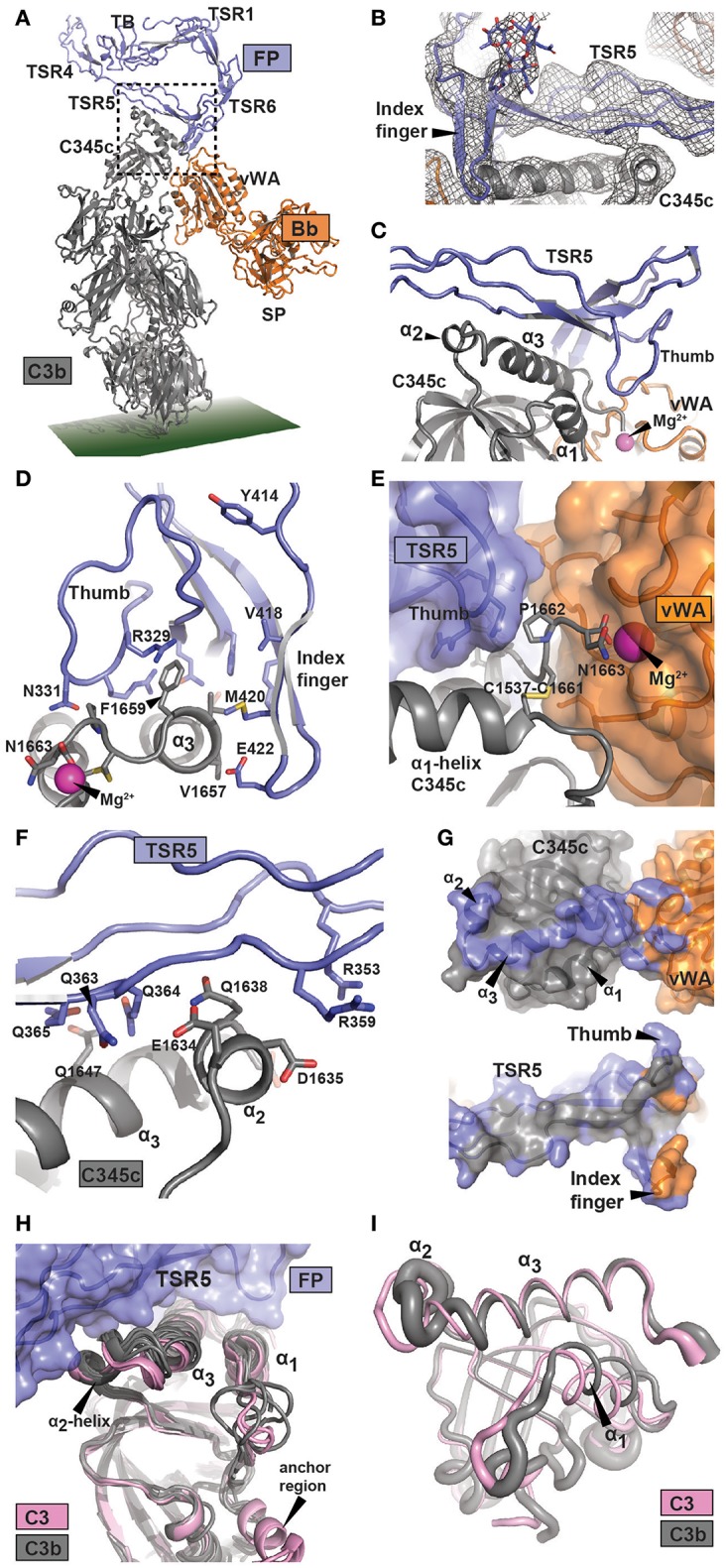
Structure of the FP bound AP C3 convertase. **(A)** Outline of the FP bound convertase shown in a cartoon representation. In FP only the TB domain, TSR1 and TSR4-6 were modeled. The SCIN protein used to stabilize C3bBb is not shown. **(B)** A 2mF_o_-DF_c_ omit map contoured at 1σ in which FP TSR5, the FP TSR6 index finger, and all associated glycosylations were omitted from map calculation. Unambiguous density is observed for the index finger and its Asn-linked glycan. **(C)** Close-up of the region outlined in panel A with a dotted rectangle containing the FP-C3b interface. The α_2_- and α_3_-helices and their connecting loop in the C3b C345c domain are recognized by the concave face of FP TSR5. **(D–F)** Details of the FP-C3 convertase interface with selected residues shown as sticks. **(D)** Outline of the interaction between the thumb and index finger of TSR5 and TSR6, respectively, and the α_3_-helix of C3 C345c. The C-terminal of C3 C345c protruding from the α_3_-helix completes the coordination of the Mg^2+^-ion present in the MIDAS of Bb. **(E)** The Bb vWA-C3 C345c interface, suggesting the importance of the FP TSR5 thumb in stabilizing the interaction. **(F)** The interaction surface formed between TSR5 and the C3 C345c α_2_ and α_3_ helix. A large number of basic residues in TSR5 are presumably interacting with the acidic α_2_ helix in C3b. **(G)** Footprint of FP on the AP C3 convertase (Top) or AP C3 convertase on FP (bottom). The footprint of FP on C3b and Bb is colored blue, whereas the footprint of C3b and Bb on FP is colored gray and orange, respectively. **(H)** Superposition of six C3b structures (gray) and two C3 (pink) structures on the central β-sheet of the C3 C345c domain. This demonstrate a clear difference around the α_2_-helix which may explain the specificity of FP for C3b. **(I)** Residue fluctuations of the C345c domain of C3b (gray) and C3 (pink) during molecular dynamics simulations. The thickness of the residues is proportional to the root mean square fluctuations in a 1 μs simulation.

We previously presented (12) a molecular envelope of FP based on omit density calculated from diffraction data extending to 6 Å from crystals containing the complex between FPc and the AP C3 convertase C3bBb and the small *Staphylococcus aureus* complement evasion protein SCIN (38). As previously described, the catalytic Bb subunit attaches to C3b through a Mg^2+^ dependent interaction in which the very C-terminal end of C3b is recognized by the von Willebrand type A (vWA) domain in the catalytic subunit Bb. Two molecules of SCIN stabilizes a convertase dimer tightly packed around a 2-fold rotation axis by simultaneously interacting with multiple regions in both C3b and Bb (Supplementary Figures 4A,B). In contrast to the spontaneous dissociation of the C3bBb convertase occurring within minutes, this dimeric SCIN stabilized convertase is stable for weeks but also incapable of binding substrate and turn over C3 (38). To obtain an improved understanding of the FP-C3bBb interaction we here also obtained diffraction data extending to 6.2 Å resolution for the complex between C3bBb, SCIN, FPcΔ3, and the FP specific non-inhibitory nanobody hFPNb1 (Supplementary Figures 4C,D). Although the resolution was low for both FP-convertase structures, the quality of the electron densities calculated without FP contribution was good due to high solvent contents, 2-fold non-crystallographic symmetry and multi crystal averaging between the two crystal forms. We then fitted the relevant parts of the FPc structure into the electron densities of both C3bBb-SCIN complexes (Figures 2A–C and Supplementary Figures 4A–D) and the structure of hFPNb1 determined at 1.3 Å resolution into the hFPNb1-FPcΔ3-C3bBb-SCIN complex (Supplementary Figures 5A–C). After this, minor rebuilding and refinement was performed to obtain the final models. For the FPc-C3bBb-SCIN complex (Supplementary Figures 4A,B) we observed weak electron densities for FP TSR2 and TSR3 and for this reason we did not model them. With respect to the hFPNb1-FPΔ3-C3bBb-SCIN structure (Supplementary Figures 4C,D), TSR2 could be modeled in one of the two complexes (Supplementary Figure 4H). The epitope of the hFPNb1 nanobody appears be located within residues 267–281 at the end of FP TSR4 located close to TSR5 and the TB domain, and all three complementarity determining regions (CDRs) seem to contribute to the paratope (Supplementary Figure 5A). In accordance with its lack of inhibition of the alternative pathway in C3 deposition assays (Supplementary Figure 5D), hFPNb1 is located 25 Å from the C3b and 58 Å from Bb Supplementary Figures 4C,D). One proof of the reliability of our convertase-FP structures was obtained when we mutated three residues in the hFPNb1 CDR3 predicted from the hFPNb1-FPcΔ3-C3bBb-SCIN structure to be important for FP recognition. Affinity measurements for the FP-hFPNb1 interaction performed with bio-layer interferometry (BLI) demonstrated that hFPNb1 had been placed in an overall correct position in the electron density at 6.2 Å resolution (Supplementary Figure 5E).

There are small differences between the four copies of the C3bBb-FP complex present in the two crystal forms (Supplementary Figure 4G). They differ by 3° rotation of the Bb von Willebrand type A (vWA) domain relative to C3b and by 7° rotation of FP relative to C3b, but crystal packing may contribute. Due to the resolution of these structures, the interactions discussed below must be considered putative, but at an overall level the model is likely to be reliable, especially due to the resolution of the input models (2.8 Å for FPc, 1.3 Å for hFPNb1, and 3.9 Å for C3bBbSCIN). We present here the C3bBbSCIN-FPc structure, and as we previously suggested (12) the major FP binding site is located within C3b residues Glu1634-Phe1659 encompassing the small α2 helix (1,634–1,638) and following large C-terminal α3 helix (1,640–1,659) in the C345c domain of C3b. Apparently there is also a smaller contribution to the convertase FP site from the Bb vWA domain. These C3bBb elements are recognized by the concave face of FP TSR5 including the “thumb” loop together with the TSR6 “index finger” loop (Figures 2D–G, Supplementary Figures 2B–D, 4E,F). One major FP-C3b contact occurs close to Bb and appears to involve a mixture of polar and non-polar interactions. A saddle shaped surface formed by TSR5 including the thumb loop and the TSR6 index finger surrounds the surface exposed non-polar C3b side chains of Val1657, Val1658, and Phe1659 (Figure 2D, Supplementary Figures 2B–D and Supplementary Table 3). Direct FP-Bb interactions appear to be limited but the strictly conserved FP Arg329 side chain may form contacts with the main chain of Bb Leu349 while the Bb side chain Tyr317 appears to reach out toward Met420 and Glu422 in the TSR6 index finger. Since the coordination of the Mg^2+^ ion in the Bb metal ion dependent adhesion site (MIDAS) by the C-terminus carboxylate from C3b is crucial for the C3b-Bb interaction, FP mediated stabilization of the AP C3 convertase may be explained by the observation that the three C-terminal residues of C3b Cys1661-Asn1663 are sandwiched between the FP TSR5 thumb loop and Bb (Figure 2E). The second major point of FP-C3b contact appears to be mediated mainly by electrostatic and other polar contacts and involves the C3b α2 helix and the N-terminal residues in the α3 helix. Specifically, this interface contains FP residues organized around the side chain of Trp318 including Arg353 and Arg359 and polar C3b residues located between Glu1634 and Gln1647 (Figure 2F). These polar C3b residues include multiple negatively charged side chains, and the electrostatic potentials of the interacting C3b and FP surfaces suggest that ionic interactions play a major role at this contact point. In summary, the FP binding site on the AP C3 convertase is dominated by C3b residues located in two separate patches. The C-terminal Asn1663 in C3b that coordinates the Mg^2+^ ion in the Bb MIDAS is likely to be considerably less mobile in the presence of the thumb loop from FP TSR5. In combination with the few putative direct FP-Bb contacts, such stabilization may underlie the reduced rate of Bb dissociation from C3b in the presence of FP.

An FP-convertase model should rationalize why native C3 does not bind FP despite that the binding site is located entirely in the C3b C345c domain, which at first glance has a very similar structure in C3 and C3b. We superimposed this domain from six C3b containing structures and two structures of native C3 including a new structure of C3 bound to the hC3Nb1 nanobody (Supplementary Table 2) on residues 1,544–1,610. This revealed a striking difference in the α2-helix and the loop connecting it to the α3-helix (Figure 2H). According to our convertase-FP structure contacts from this region are likely to be formed with FP TSR5 residues Arg351-Arg359 (Figure 2F), and if the C3 conformation of this region was adopted these contacts could probably not be formed. The α3 helix as such is also slightly rotated when comparing C3b and C3, resulting in a small difference between the two states in the location of Phe1659 at the heart of the non-polar C3b-FP interaction.

We also investigated whether the dynamic properties of the FP binding regions differed between C3 and C3b by conducting molecular dynamics simulations of the two full proteins (Figure 2I and Supplementary Figure 6). A comparison of the dynamic properties of the putative FP binding regions revealed a clear difference between the two states of the C345c domain, especially around residues 1,580–1,595, 1,609–1,615, and 1,635–1,645 including the α2-α3 region presumably involved in FP binding (Supplementary Figure 6A). Altogether, our analysis indicates that the FP preference for C3b is mainly due to a conformational difference in and around the C345c α2-helix supplemented with an increased mobility of this region. These changes may originate from the anchor region located immediately prior to the C345c domain which undergoes a conformational rearrangement in terms of both location and secondary structure upon conversion of C3 to C3b (39). Importantly, the high overall mobility of the C345c domain in C3b was confirmed by our simulation (Supplementary Figure 6B) reproducing a well-established property of this domain observed in crystal structures. This mobility of the domain may facilitate its search for a C3b binding site in a nearby FP molecule.

### Validation of the FP-Convertase Interactions

**Figure 3:**
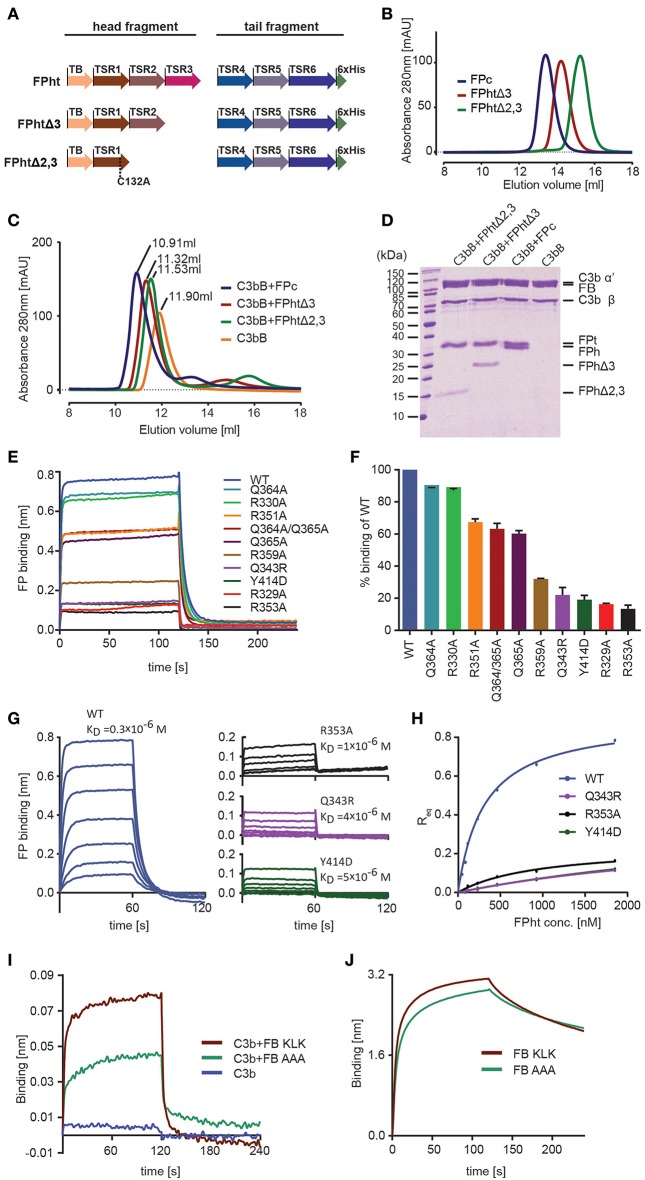
Validation of the FP-C3 AP convertase and definition of a minimal FP fragment. **(A)** monomeric FP head-tail (ht) proteins created by co-expression from two open reading frames. **(B)** Chromatograms from analytical SEC analysis of FPc (blue), FPhtΔ3 (red), FPhtΔ2,3 (green). **(C)** SEC analysis of AP C3 proconvertase formation using FPc (blue), FPhtΔ3 (red), FPhtΔ2,3 (green), or no FP (orange) demonstrating that all the tested FP monomers formed a stable complex with the proconvertase. **(D)** SDS-PAGE analysis of the peak fractions from the SEC runs in panel C. **(E)** Sensograms showing binding of WT and mutated FPht to a C3b coated sensor. **(F)** Quantification of FPht binding expressed in % of the WT signal. The signal corresponds to the average signal taken from 80 to 120 s in two independent experiments. The standard deviation is represented by error bars. **(G)** Sensograms obtained in concentration series ranging from 1,852 to 29 nM for FPht, FPht R353A, Q343R, and Y414D. The K_D_ obtained from steady state analysis of the data is indicated in the figure. **(H)** Steady state analysis of the data shown in panel G. The measured R_eq_ (average signal from 40 to 60 s) is shown as a function of FPht concentration. **(I)** Sensograms for binding of C3b, C3bB KLK, and C3bB AAA to a FP coated sensor. The C3bB KLK complex binds more strongly to FP than C3bB AAA. **(J)** Sensograms for binding of FB KLK and FB AAA to a C3b coated sensor demonstrating that the effect of the FB AAA mutation shown in panel I is not due to decreased C3b binding.

Identification of the FP regions mediating oligomerization in our structures led us to hypothesize that a monomeric FP equivalent to FPc could be assembled correctly in mammalian cells if the TB-TSR1-TSR2-TSR3 head and the TSR4-TSR5-TSR6 tail fragments were expressed as separate polypeptides (Figure 3A). As our structures did not indicate any role for TSR2 and TSR3 in FP oligomerization and convertase interaction, we also deleted TSR2-TSR3 or TSR3 from the head fragment and observed that complexes between the intact tail fragment and these two truncated head fragments indeed were secreted. The resulting purified FPhtΔ3 and FPhtΔ2,3 were active in forming stable complexes with the proconvertase C3bB in size exclusion chromatography (Figures 3B–D). In the light of these data and our structures of the FP-convertase complex we conclude that FPhtΔ2,3 is the minimal FP monomer that is functional in convertase binding. These results are in agreement with the fact that active oligomers are formed by FP lacking TSR3 (19).

The head-tail expression system only generates FP monomers and therefore uncouples the effect of FP mutations from oligomer distribution. This is important as FP activity in complement activation assays and sensor-based interaction measurements crucially depends on the oligomer distribution (12). We therefore used FP variants produced in the head-tail system with 1–2 residues mutated to alanine to validate the contacts between FP and C3b inferred from our convertase structure and the human FP deficiency mutants Q343R and Y414D (see below). All FPht variants were purified to high homogeneity and did not show signs of aggregation suggesting proper folding. In BLI experiments with immobilized C3b all the FPht mutations resulted in decreased binding to C3b (Figures 3E,F). Severe defects were observed for Q343R, Y414D, R329A, and R353A. Wild type (WT) FPht had a dissociation constant K_D_ = 0.3 μM for C3b, mutation of Arg353 increased this to 1 μM whereas the two FP deficiency mutants gave K_D_ values of 4–5 μM. (Figures 3G,H).

Based on the proximity of FP TSR5-6 to the Bb vWA domain and FB sequence conservation we also prepared a variant with 348-KLK-350 mutated to alanine in a FB molecule already carrying the D279G mutation giving stronger C3b binding (40) and S699A rendering it proteolytically inactive. We then bound WT FPht to a hFPNb1 coated BLI sensor and measured binding of C3b and the AP C3 proconvertases assembled with the FB variants 348-KLK-350 and 348-AAA-350. Significantly less binding of the C3bB AAA proconvertase as compared to the C3bB KLK was observed (Figure 3I), supporting a role of these residues in the FB-FP interaction within the AP C3 proconvertase. The decreased binding of FB AAA is not due to decreased formation of the C3bB proconvertase itself, because when we measured binding of FB KLK and FB AAA directly to immobilized C3b no significant differences between the two FB variants were observed (Figure 3J). Altogether, these biophysical data supports our structure-based mapping of the C3b interacting residues in FP and provides further evidence for a direct FP-FB interaction in the proconvertase. In combination with our ability to identify the hFPNb1 residues in contact with FP, these results also confirm the reliability of our X-ray structures of convertase-FP complexes.

### FP Inhibits FI-FH Mediated Degradation of C3b to iC3b

**Figure 4:**
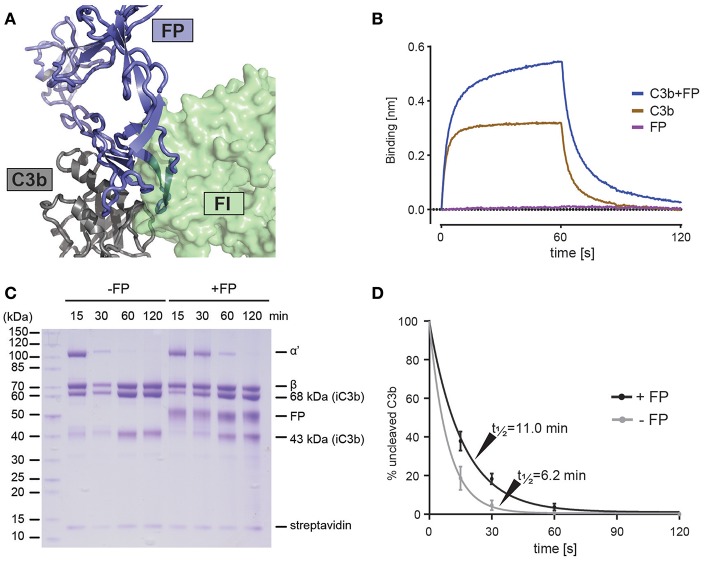
FP competes with FI but not FH for C3b binding. **(A)** Superposition of the C3bBbFP structure and the C3b-FH-FI structure (PDB entry 5O32), showing that FI and FP have overlapping binding sites on surface of the C3b C345c domain. C3b is shown in gray, FI is shown in green and FP is shown in blue. **(B)** BLI binding experiment demonstrating that FP and miniFH can interact with C3b simultaneously. The sensograms for the association phase (0–60 s) and dissociation phase (60–120 s) are shown for the interaction of a miniFH coated sensor with either C3b (brown), FP (red) or C3b preincubated with FP (blue). **(C)** SDS-PAGE analysis of FH-assisted FI proteolysis of biotinylated C3b on streptavidin beads either in the presence (+FP) or absence (–FP) of FP. Conversion of C3b to iC3b is observed to be decreased in the presence of FP. **(D)** A quantification of the assay shown in panel C. The ratio between the intensities of the α' and streptavidin bands from three independent experiments is used as a measure of uncleaved C3b. Error bars indicate the standard deviation between the three experiments. The cleavage was fitted with a first-order exponential decay.

Cofactor dependent degradation of C3b by factor I (FI) represents an important regulatory mechanism of the alternative pathway on host tissue (41). Factor H (FH) is a soluble cofactor that through interaction with surface glycan allows C3b degradation by FI, reviewed in Blaum (42). Farries et al. showed that the presence of FP inhibited FI interaction with erythrocyte bound C3b and made erythrocyte lysis less sensitive to FI, indirectly suggesting that FP inhibits C3b degradation due to FI-FP competition as an additional mechanism for stimulating AP activity (43). Our structures suggests a strong overlap between the binding sites for the FI FIM domain (44) and FP on the C3b C345c domain (Figure 4A). To analyze the FP-FI competition in a minimal system we immobilized mini-FH (22) on a BLI sensor to confirm that C3b can bind mini-FH and dimeric FP simultaneously (Figure 4B). Next, we investigated the effect of FP on FH-assisted FI degradation of C3b in a minimal system where we immobilized C3b on streptavidin beads and followed its FI degradation to iC3b with FH as cofactor (Figures 4C,D). In the presence of a mixture of trimeric and dimeric FP, we observed a significantly slower formation of iC3b in agreement with a direct competition between of FP and FI. These and prior data (43) confirm the importance of the interaction between the FI FIM domain and the C3b C345c domain (44) and are consistent with the observed effects of membrane cofactor protein on C3bBb decay and C3b degradation in the presence of FP (18).

### The FP Oligomerization Interfaces

**Figure 5:**
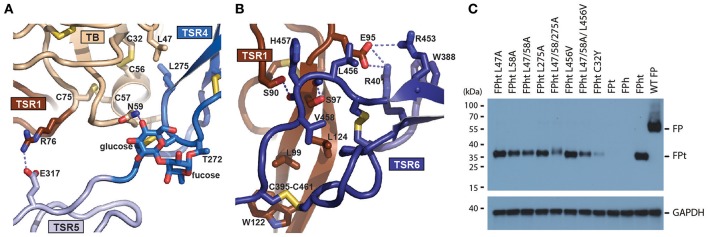
Structure of the FP oligomerization interface and analysis of its stability. **(A)** Outline of the interface between TB and TSR4 of FP. Important residues are shown in an all-atom stick representation. The O-linked glycan on T272 is directly involved in the interaction of TSR4 with the TB domain through a hydrogen bond (dotted line). **(B)** As panel A but presenting the interface between TSR1 and TSR6. **(C)** Western blot analysis of cell supernatants obtained with different FPht mutants. To account for differences in total amount of cells a western blot of GAPDH from the cell pellet was performed using the same amount of sample volume.

Our structures offer structural insight into how FP oligomers are maintained through two major contacts between the TB-TSR1 from one monomer and TSR4-6 from a second monomer. The TB domain recognizes the TSR4 domain from another monomer while the concave face of TSR1 from one monomer interacts with a short C-terminal extension of TSR6 from the second monomer (Figures 5A,B). Analysis of the interface between the two chains reports a total interface of 1,109 Å^2^ with 665 Å^2^ for the TSR1-TSR6 interface and 444 Å^2^ for the TB-TSR4 interface. The TSR1-TSR6 interface is also the more hydrophobic according to PISA (45). The TB-TSR4 interface is centered on Leu58, Cys312, and Leu275 with smaller contributions from Leu47, Phe62, and Ile305. Nearby, a salt bridge between Arg76 and Glu317 links the TB domain with TSR5. The TSR1-TSR6 interface is centered on the contacts formed by Pro399 with Leu124, the stacking of the Trp122 with the Cys395-461 disulfide and hydrogen bonds between Ser97-Leu456 and Ser90-His457. To validate the two oligomerization interfaces, we established a cellular assay taking advantage of the fact that secretion of the tail fragment depends strictly on the expression of the head fragment. Hence, secretion of FPht variants could be followed by detection of the His-tag present on the FP tail. To challenge the non-polar contacts observed between the TB domain and TSR4 we mutated Leu47, Leu58, and Leu275 to alanine. Mutation of Leu58 and Leu275 had significant effects and in the triple mutant secretion was severely affected (Figure 5C). Mutation of Leu456 to valine at the TSR6-TSR1 interface had a modest effect in this assay despite its coupling to inflammatory bowel disease (46) whereas there was a severe defect in FP secretion for the triple mutant L47A/L58A/L456V affecting both the TB-TSR4 and the TSR6-TSR1 interaction (Figure 5C). Overall, these mutations confirmed the two observed oligomerization interfaces.

### Naturally Occurring FP Mutants Disrupting Function

Our results offer a structure-based explanation for lack of AP activity caused by FP deficiencies. In type I deficiency there is no FP secreted most likely due to intracellular quality control triggering degradation of truncated or misfolded FP. The mutations G298V, W321S/G, and R346C cause type I deficiency (5, 47, 48). The presence of a valine side chain instead of Gly298 would prevent insertion of Trp260 in the Trp/Arg stack of TSR4 possibly leading to incorrect folding of TSR4 and a decreased stability of the TB-TSR4 interaction in the oligomer. Disruption of the Trp stack in TSR5 is also a likely consequence of the Trp321Ser/Gly as well as the mutation Arg346Cys.

**Figure 6:**
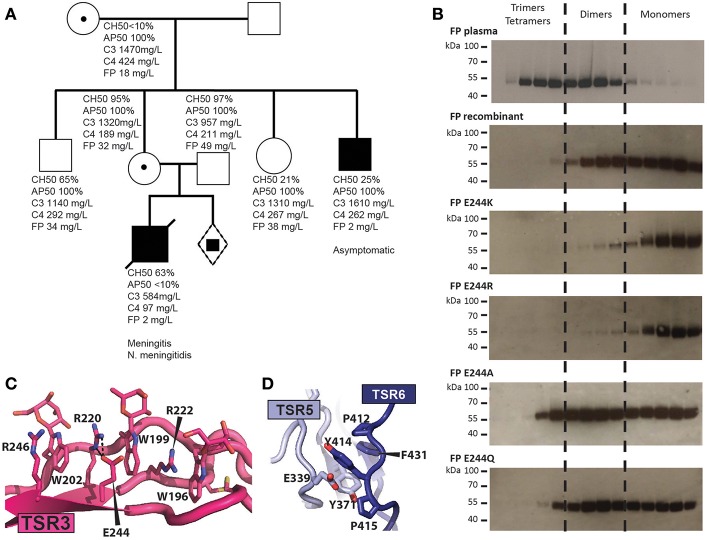
Molecular basis for naturally occurring FP deficiencies. **(A)** Pedigree of the FP C32Y deficiency family. Plasma FP levels for the patient were below 10% of normal. AP50 and CH50 were determined for the son and showed normal CP but impaired AP activity. The FP level of his heathy uncle who carries the FP pathogenic variant was below 10% of normal value without detectable impairment of the CP and AP activities. His father as well as the heterozygous mother and grandmother both carrying the FP mutation exhibited normal complement activity profiles (AP50/CH50) and FP serum levels. **(B)** Oligomer distribution obtained with WT FP and variants carrying a mutation at residue 244. Recombinant FP was affinity purified on hFPNb1 beads, fractionated by SEC and the resulting fractions were analyzed by western blotting. **(C)** Outline of the TSR3 tryptophan-arginine stack demonstrating how glutamate 244 and Arg220 interact electrostatically. **(D)** Close-up view of Y414 and surrounding residues neighboring the thumb and index finger in TSR5 and TSR6, respectively. Y414 is stacking between E339 and P412 and is located in a hydrophobic pocket harboring F431 and Y371.

In type II deficiencies the FP serum level is abnormally low and oligomerization is affected. We identified a novel type II FP deficiency in a 1-year old boy who was hospitalized after 2 days of abdominal pain, vomiting, severe neurological deterioration and appearance of purpuric skin eruption associated with fever. Two days after the onset of the first symptom, the patient was admitted to the emergency department with hemodynamic instability with tachycardia, severe hypotension together with an extensive and necrotic purpura. Despite massive IV fluids and adrenaline administration, the patient died 24 h after admission. Blood culture and skin biopsies came back positive for *Neisseria meningitidis* serogroup W135. A screening for FP deficiency revealed one asymptomatic uncle. The mother, the grandmother and a still unborn child carried the genetic trait of the pathogenic allele (Figure 6A). The causative Cys32Tyr mutation disrupts one of the three disulfide bridges in the TB domain which may lead to a defect in the TB-TSR4 head-tail interaction due to the vicinity of Cys32 to TSR4 (Figure 5A). Analysis by head-tail expression also showed very low levels of secreted FP C32Y confirming this model (Figure 5C).

We previously described how another type II deficiency caused by the Glu244Lys mutation in TSR3 results in very low AP activity, and recombinant FP E244K is secreted almost exclusively as a monomer with a compact structure (12). Our FPc crystal structure reveals that Glu244 interacts electrostatically with Arg220. To analyze whether the charge reversal of glutamate to lysine underlies the altered oligomer distribution we compared the oligomer distribution for three additional mutations to those of WT FP and FP E244K. The E244R variant was like FP E244K and was secreted almost exclusively as monomers, while mutation to alanine, or glutamine resulted in an oligomer distribution resembling that of wild type recombinant FP (Figure 6B). This suggests that charge reversal mutations at residue 244 destabilizes Arg220 and thereby presumably the Trp/Arg stack containing Arg220 (Figure 6C). This most likely translates into incomplete folding and degradation explaining the low abundance of FP in the plasma of E244K carriers (12). The failure of FP E244K oligomer formation is more difficult to rationalize from the structure.

Another example of type II deficiency is the Arg100Trp substitution where a tryptophan takes the place of an arginine in the TSR1 Trp/Arg stack. This substitution may weaken the nearby TSR1-TSR6 interaction in one of the two oligomerization interfaces (Figure 5B) rationalizing the low serum level and altered oligomer distribution (49). Mutation of Gln343 to Arg also results in type II deficiency with a shift in the oligomer distribution toward dimers (49). Gln343 is located at the C3b binding site and our binding studies showed that its mutation to the larger arginine significantly decreases the FPht affinity for C3b (Figures 3E–H). There is one known type III deficiency, the Tyr414Asp mutation where serum FP level and oligomer distribution is normal but AP activity is absent and FP binding to immobilized C3bB and C3bBb is very low (50). Using BLI, we confirmed a decreased FPht Y414D affinity for C3b (Figures 3E,H). The structure rationalizes these results as Tyr414 is located immediately before the index finger loop in TSR6 and neighbors the thumb loop in TSR5, and both loops are part of the C3b binding site and also face Bb (Figure 6D). A mutation to aspartate is likely to lead to repulsion between an aspartate in position 414 and Glu339 and thereby disturb the structure of the index finger loop involved in convertase interaction. In summary, our structures of FP and the FP-convertase complex enable an understanding of the functional defects associated with novel and known FP deficiencies not caused by premature stop codons.

## Discussion

The atomic structures presented here represents a quantum leap in our understanding of how the function of FP relates to its structure. Although electron microscopy and our first crystallographic study clearly revealed the eye-shaped vertex interacting with the C3 convertase, essential questions including which TSRs form the vertex, what is the fold of the N-terminal region, how do two FP molecules come together and form the vertex structure, and which structural features allow FP to form a spectrum of oligomers could not be answered (12, 18). All these questions are now settled by our structures.

### FP Stimulation of the Alternative Pathway

The primary biological function of FP is stimulation of AP convertase activity and our structures now unambiguously define the C-terminal domain of C3b as the primary binding site supplemented with a contribution from Bb. A far more extensive interaction of FP with the Bb vWA domain inferred from negative stain EM (18) is not supported by our structures. In our first crystallographic study we presented an electron density for the core part of FP at 6 Å resolution bound to the convertase. But in the absence of an atomic structure of FP, we could only identify in an overall manner the region in C3b interacting with FP. Furthermore, we could not identify which parts of FP that were in direct contact with C3b and Bb although we did suggest that TSR5 plays a key role (12). In the presence of the atomic structure of FPc we can now present a much improved model for the FP-convertase interaction. It is now clear that the thumb loop in FP TSR5 contributes to a binding pocket for the C-terminus of C3b and that the index finger loop FP TSR6 is likely to interact directly with FB and Bb.We also conclude based on structural comparisons and molecular dynamics simulations that the specificity for C3b over C3 is primarily due to subtle structural differences and differences in mobility in a small stretch of C3b residues involved in FP binding.

Our mapping of the convertase binding site to FP TSR5 and TSR6 agrees with the properties of FP deletion mutants (19, 51), and the epitope location of monoclonal antibodies blocking FP activity (52). A bacterially expressed fusion protein containing TSR4-TSR5 has been reported to act as an alternative pathway inhibitor (53, 54). Our structures argue that in the absence of TSR6, major parts of the C3b binding elements located in TSR5 of such a recombinant fragment will not be fully functional. Furthermore, the absence of the C- and O-linked glycans that our structures now reveal as well-ordered and interacting with protein residues also argues that experimental data obtained with bacterially expressed FP fragments should be interpreted with caution.

Our structural and functional studies also emphasize that FP contributes in at least three ways to enhance the activity of the alternative pathway. First, there is a synergistic recruitment of FP and FB to the AP proconvertase (12, 55, 56), but the structural basis for this remains to be clarified, possibly there are additional contacts between FP and FB compared to the FP-convertase complexes. Data presented here show for the first time that contacts between FP and the FB vWA domain are important for FP stimulated formation of the proconvertase (Figures 3I,J), but comparison with the structure of the FD bound proconvertase (57) also suggests that the CCP1 domain in FB may form direct contacts with the thumb loop in FP TSR5. Second, our structures suggest that the slower dissociation of the C3bBb complex in the presence of FP is due to a tighter binding of the C3b C-terminus to the Bb MIDAS supplemented with a few direct FP-Bb interactions. Here the thumb loop in TSR5 appears to play a key role as it together with the vWA domain in Bb forms a binding pocket for the C-terminal residues in C3b. Third, our functional data and structural comparisons (Figure 4) conclusively show that FP compete directly with FI and thereby counteracts the most important mechanism for negative regulation of the alternative pathway in which FH and other complement regulators by binding to C3b create a binding platform for FI (44, 58) enabling the irreversible C3b degradation. FP does not directly compete with mini-FH (Figure 4B), but by stabilizing both the proconvertase and the convertase it will indirectly make it harder for FH and other regulators to dissociate FB and Bb from C3b.

Negative stain EM suggested that all the convertase binding sites in oligomeric FP could bind a convertase simultaneously (18) in agreement with the dimer model presented in Figure 1F, where the two convertase binding sites are located at each pole of the dimer. The activity of FP oligomers in erythrocyte lysis follows the order tetramers > trimers > dimers (11), and the FPc monomer is much less active in convertase stabilization on erythrocytes and bactericidal activity compared to oligomeric FP (12). We cannot exclude that the convertase binding site we identify encompassing the concave face of TSR5 and the index finger loop from TSR6 has a slightly different structure in oligomeric FP. However, the flexibility enabling FP to form the different oligomers argues that there is no allosteric communication between convertase binding sites within a single oligomeric FP molecule, and we suggest that the convertase binding sites are independent. The higher activity of oligomers could have contributions from, (i) A single FP oligomer binding multiple activator-bound C3b molecules, (ii) a high local concentration of C3b binding sites favoring FP rebinding after dissociation, (iii) a defined 3D structure of the oligomers. Our prior SPR data showed a 450x lower apparent K_D_ value for immobilized C3b and fluid phase oligomeric FP as compared to the reverse geometry (12) arguing for a contribution from multivalent binding. In contrast, binding experiments measuring FP association to erythrocyte bound C3bB and zymosan-C3b complexes suggested that FP oligomers bind to a C3b coated activator in a monovalent manner (43, 55). A better understanding on how FP oligomerization contributes to activity may require comparison of activity in multiple functions of natural FP oligomers with engineered oligomers formed by multimerization of FP monomers by other means. The atomic structures of FP also opens new opportunities for developing much improved models of the FP oligomers based on data from solution scattering and other biophysical techniques compared to prior models developed previously (17) that could not take advantage of the detailed atomic structures presented here.

### Role of FP in C5 Convertases

An unresolved question concerns the role of FP for the activity of C5 convertases. These are formed from the alternative pathway C3 convertase C3bBb and the homologous classical pathway C3 convertase C4b2a at a high density of C3b on the activator. The C5 convertases initiates the terminal pathway where cleavage of complement C5 results in release of the potent proinflammatory anaphylatoxin C5a that activate a range of cell types through binding to the C5aR1 receptor (59). Furthermore, C5b is generated and nucleates the assembly of the membrane attack complex that may lyse pathogens but also host cells with defects in complement regulation as exemplified by the lysis of red blood cells in paroxysmal nocturnal hemoglobinuria (60). The exact composition and structure of the C5 convertases are not clear, it remains to be settled whether there is an additional C3b molecule directly associated with both C5 convertases or the role of the high C3b density is to recruit and prime C5 for cleavage by a C3 convertase, reviewed in Schatz-Jakobsen et al. (15). Since FP promotes the required high C3b density through its stimulation of the alternative pathway C3 convertase, it is not surprising that FP deficiency or blockade inhibits the terminal pathway (61). At the high C3b density required for formation of both C5 convertases, oligomeric FP must bind avidly. The FP stabilized alternative pathway C3- and the C5-convertases decays by Bb dissociation with similar kinetics (62) suggesting that FP binds to the C3bBb part within the alternative pathway C5 convertase as observed here for the C3 convertase. In contrast, FP binding to C3b has been reported to inhibit the classical pathway C5 convertase activity without interfering with CP C3 convertase activity (63) indicating that the C3b C345c domain holding the FP binding site have a function in the CP C5 convertase activity. Since none of the C5 convertases have been reconstituted in a soluble format, structural insight on the function of FP in relation to the C5 convertases may require tomograms of vesicles onto which C3b and the convertases have been deposited (64). Such studies may also lead to an improved understanding of C3 nephritic factors (C3NeFs), which are prevalent in C3 glomerulopathy (65). C3NeFs binds a neoepitope in the labile AP convertases and prolong their half-life. FP-dependent C3NeFs have been shown to stabilize C5 convertases (66) underscoring the role for FP in C5 convertase function.

### Pattern Recognition and NKp46 Recognition

In contrast to the convertase related functions, the role of FP as a C3b independent pattern recognition molecule are still unclear. FP has been proposed to bind directly to pathogens, activated platelets and apoptotic/necrotic host cells and thereby recruit fluid phase C3b or the C3 tick-over product C3-H_2_O and initiate alternative pathway amplification on the FP binding pattern, reviewed in Chen et al. (3). Recently, C3b independent recruitment of FP has been questioned (67), but in a C3 knockout mouse FP was still deposited at injured glomeruli *in vivo* although the responsible FP binding pattern was not identified (68). Molecular patterns suggested to be recognized by FP include LPS, negatively charged host glycan, zymosan, acetylated LDL, and multiple proteins. Interestingly, our structure of FPc hints at how FP mediates pattern recognition. Negatively charged sulfated groups on host glycans may form electrostatic interactions with the C-terminal end of TSR3, where two sulfate ions are firmly bound to Arg247 (Supplementary Figure 3C). In addition, the crystal packing of two TSR2 domains presented in Supplementary Figure 3B illustrates the potential of the TSR mannosylations and the surrounding residues for forming hydrogen bonds with carbohydrate groups on host glycan or glycosylated proteins. Another novel interesting function of FP is the interaction with the NK cell receptor NKp46 enabling recruitment of NKp46 presenting cells to *Neisseria meningitidis* bacteria. Mice without NKp46 positive cells are less capable of clearing this bacteria through a MAC independent pathway (69), but the molecular mechanism behind the NKp46-FP interaction remains unclear.

## Conclusions and Outlook

This study present the first detailed structures of FP and overall models of FP-convertase complexes. We also identify regions in C3b and Bb interacting with FP, and we describe a minimal FP fragment for convertase binding. Altogether, these results will greatly facilitate the design of FP variants for *in vitro* experiments and transgenic animals for the analysis of the crosstalk between FP convertase stimulation, pattern recognition and NKp46 interaction. The structures also enable a rational approach to answer fundamental questions regarding FP oligomerization that remains to be addressed. Why is it advantageous to have a polymer distribution rather than a monomeric protein for enhancing alternative pathway activity? Is the increase in biological activity with oligomer size solely due to an increasing number of FP subunits or is there a structural component? In a translational perspective, the interest in therapeutic modulation of the complement system is steadily increasing due to the association of excessive complement activation triggering inflammation and tissue damage in eyes, kidneys, skin, brain and the vasculature, reviewed (70). The successful blockade of the terminal pathway has provided proof-of-concept for therapeutic complement inhibition, but blocking of the upstream C3 cleavage by the alternative pathway convertase stimulated by FP could be an alternative strategy. There have been developed a large number of AP convertase inhibitors targeting in particular C3 and Factor D, but monoclonal antibodies acting as FP inhibitors have also been described, reviewed in Chen et al. (3) and Morgan and Harris (14). The therapeutic potential in FP inhibition has been demonstrated in animal models of arthritis, atypical hemolytic uremic syndrome and paroxysmal nocturnal hemoglobinuria (61, 71, 72). The structures of FP and its convertase complexes offer a much improved foundation for development of FP inhibitors as they pinpoint three-dimensional epitopes and pockets on FP, C3b, and FB that may be targeted for development of specific modulators of the alternative pathway.

## Data Availability

Diffraction data and atomic models are deposited at the protein data bank PDBe under the entries listed in Supplementary Tables 1, 2.

## Author Contributions

DP, TG, RJ, SM, and MR purified proteins, determined structures, and conducted functional assays. CT and NJ took care of patients. CE and VF-B performed experiments and formulated the biological diagnosis. YW and KL-L conducted and analyzed molecular dynamics simulations. SP performed mass spectrometry and analyzed data. NL selected hFPNb1. ST and GA supervised research. DP and GA wrote the manuscript.

### Conflict of Interest Statement

The authors declare that the research was conducted in the absence of any commercial or financial relationships that could be construed as a potential conflict of interest.
