# Supplementary figures and images for: Structural Basis for Properdin Oligomerization and Convertase Stimulation in the Human Complement System
Source: Front Immunol. 2019 Aug 22;10:2007. doi: 10.3389/fimmu.2019.02007 (PMC6713926; doi:10.3389/fimmu.2019.02007)

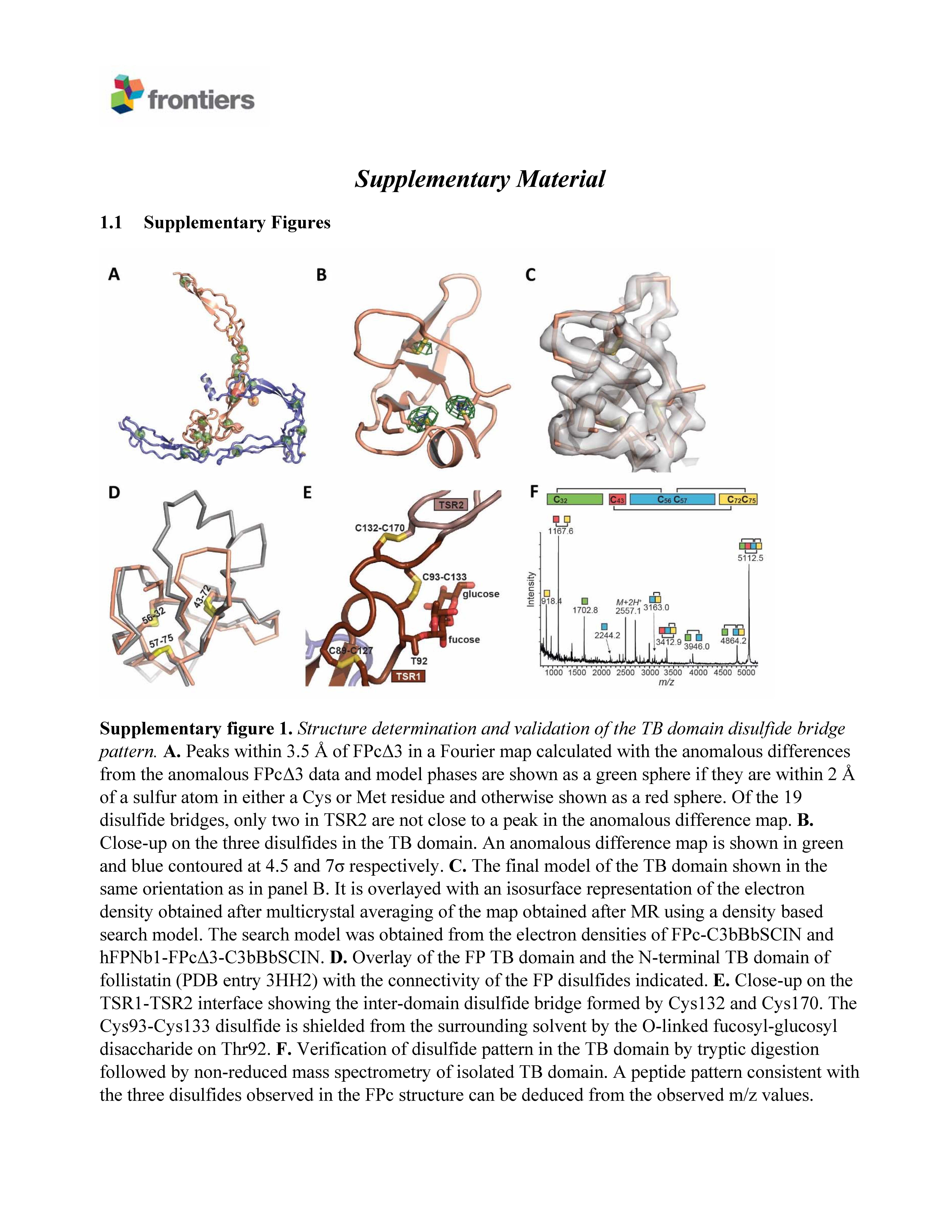

Supplement: Supplementary file 1 [file Image_1.JPEG]

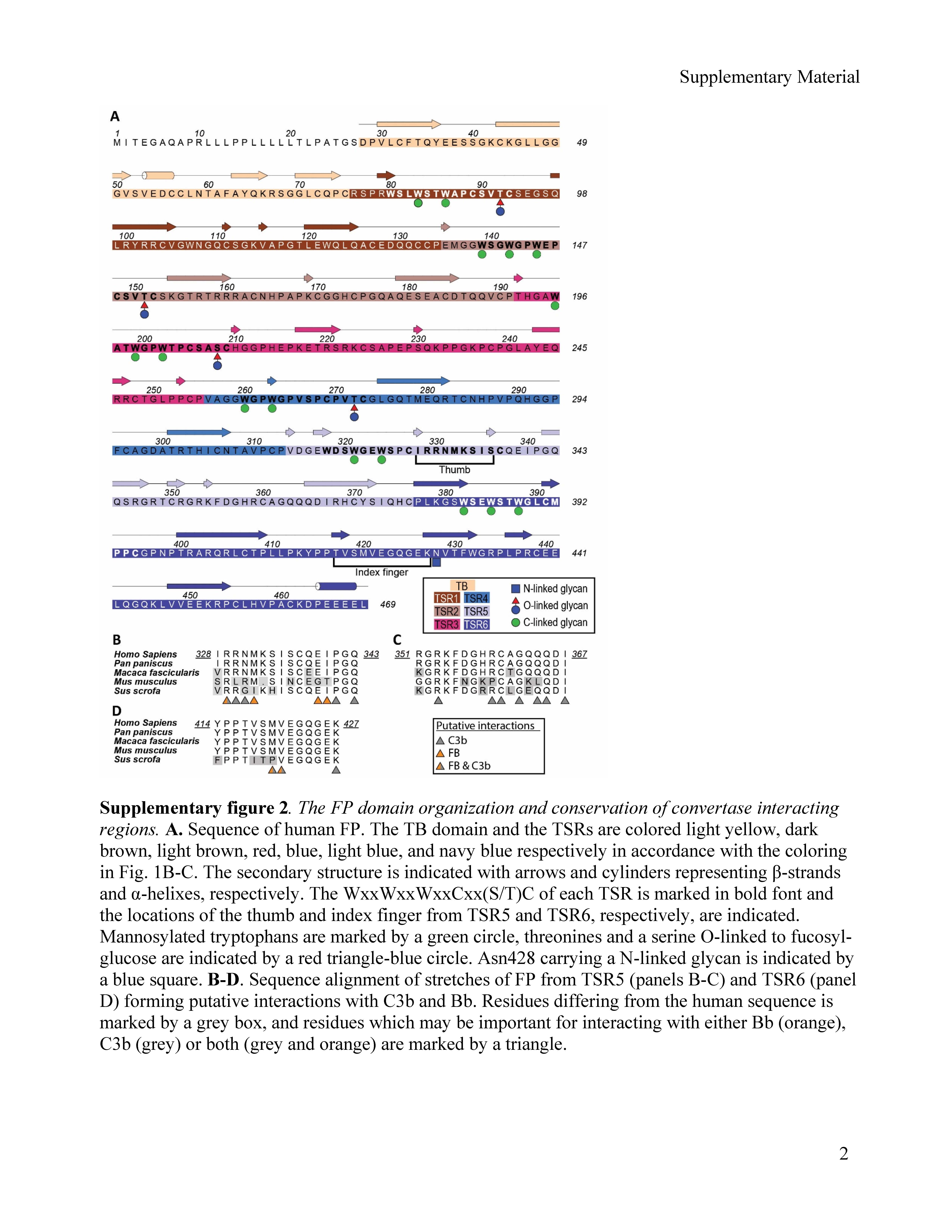

Supplement: Supplementary file 2 [file Image_2.JPEG]

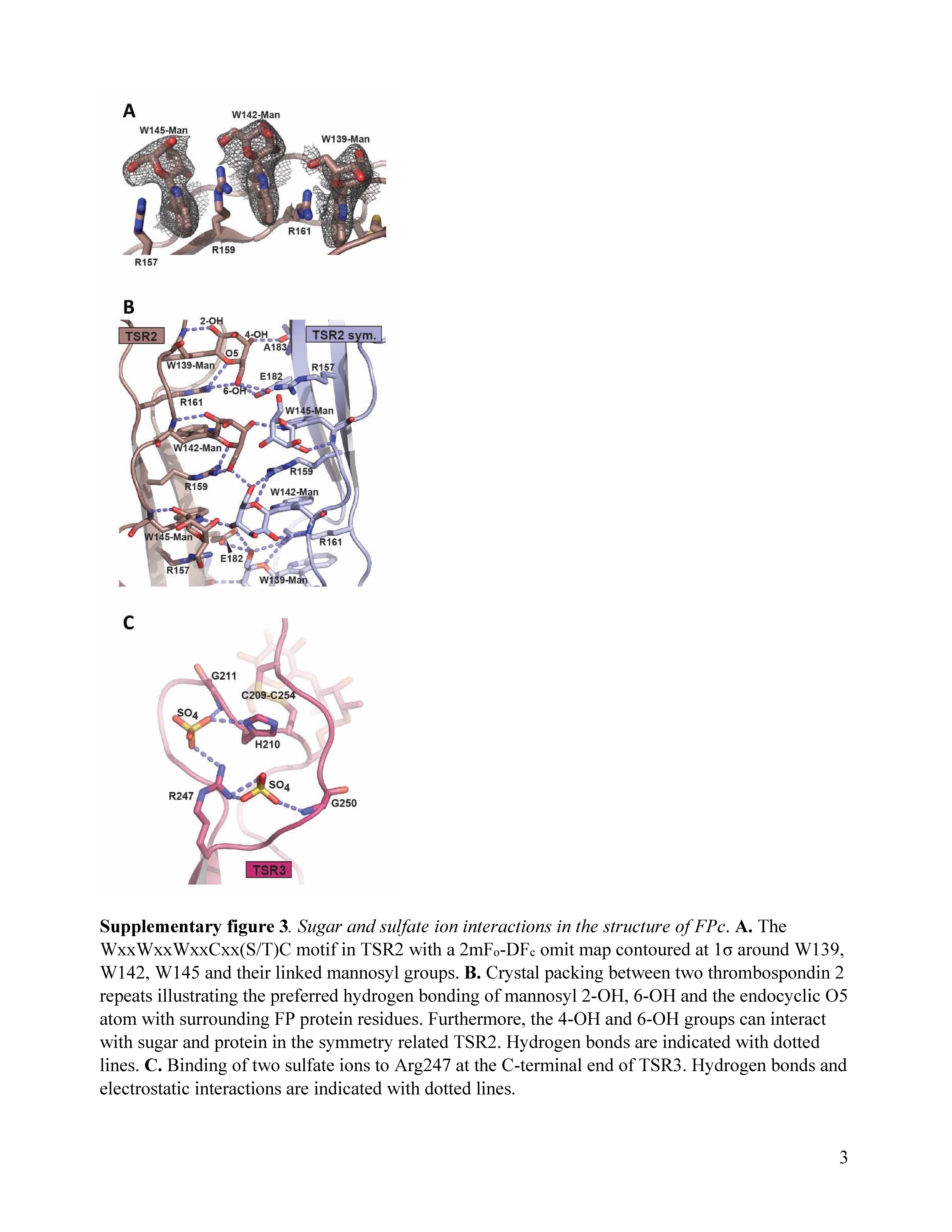

Supplement: Supplementary file 3 [file Image_3.JPEG]

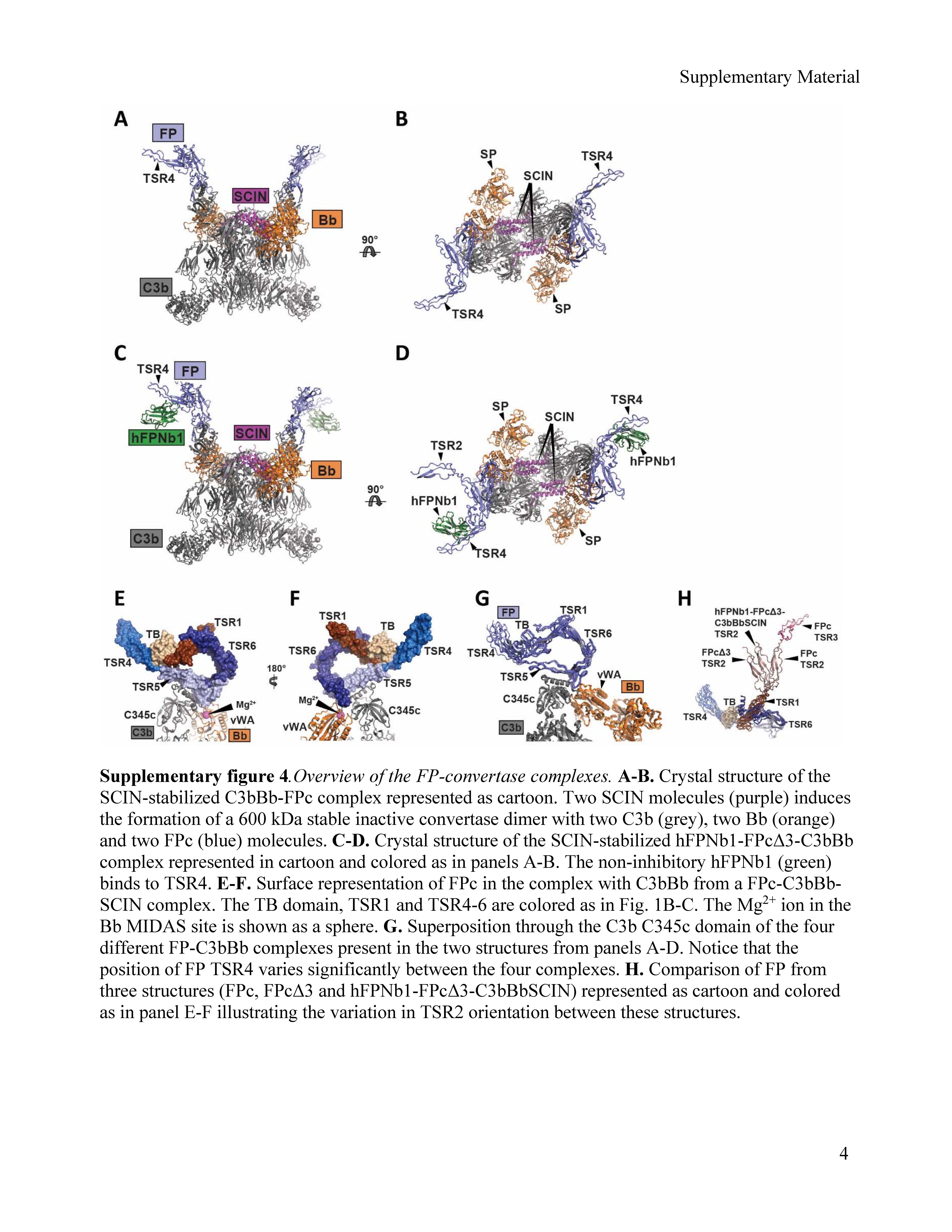

Supplement: Supplementary file 4 [file Image_4.JPEG]

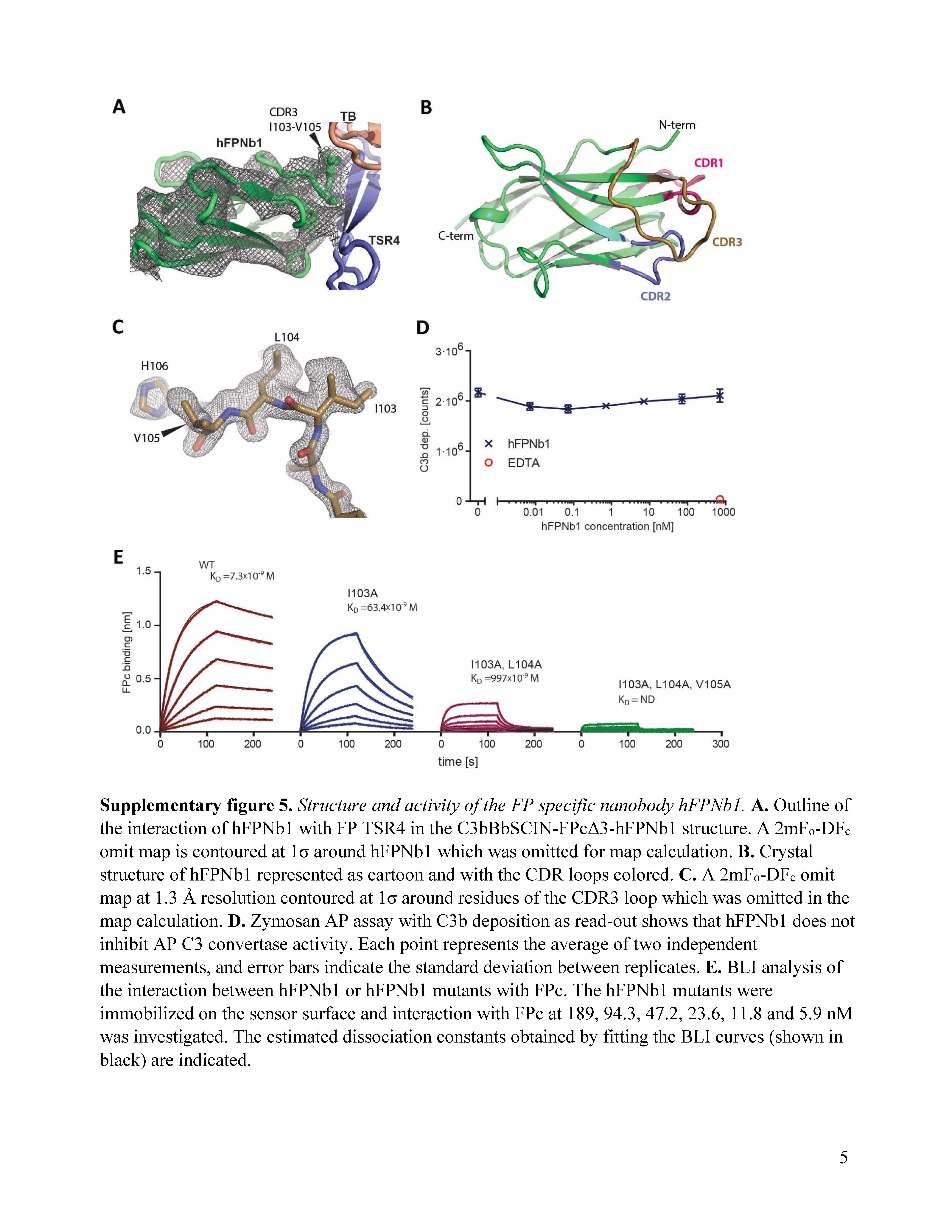

Supplement: Supplementary file 5 [file Image_5.JPEG]

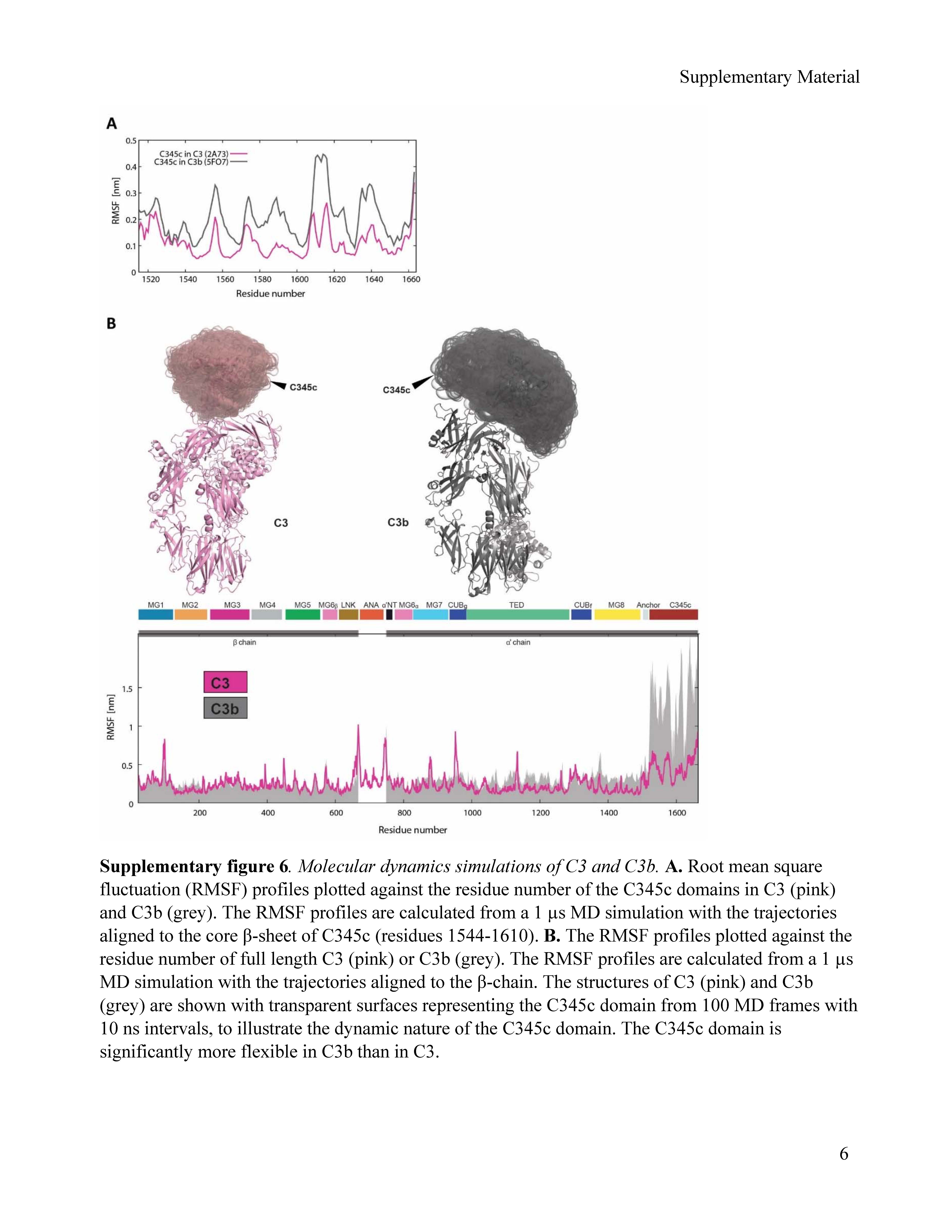

Supplement: Supplementary file 6 [file Image_6.JPEG]
