# Supplementary material for: Structural Basis for Properdin Oligomerization and Convertase Stimulation in the Human Complement System
Source: Front Immunol. 2019 Aug 22;10:2007. doi: 10.3389/fimmu.2019.02007 (PMC6713926; doi:10.3389/fimmu.2019.02007)
Supplement: Supplementary file 7 [file Table_1.DOCX]

**Supplementary table 1**. Refinement statistics for the structures of FPc, FPcΔ3 and C3bBbSIN-FPc where data collection statistics are published (Pedersen et al., 2017;Pedersen et al., 2019). Statistics for the highest-resolution shell are shown in parentheses.

Pedersen, D.V., Revel, M., Gadeberg, T.a.F., and Andersen, G.R. (2019). Crystallization and X-ray analysis of monodisperse human properdin. Acta Crystallogr F Struct Biol Commun 75, 0.

Pedersen, D.V., Roumenina, L., Jensen, R.K., Gadeberg, T.A., Marinozzi, C., Picard, C., Rybkine, T., Thiel, S., Sorensen, U.B., Stover, C., Fremeaux-Bacchi, V., and Andersen, G.R. (2017). Functional and structural insight into properdin control of complement alternative pathway amplification. EMBO J 36, 1084-1099.

| **Structure** | **FPc** | **FPcΔ3** | **C3bBbSCIN-FPc** |
| --- | --- | --- | --- |
| PDB entry | 6RUS | 6SEJ | 6RUR |
| Resolution | 45.6-2.8 | 49.7-3.5 | 48.8-6.0 |
| Reflections used in refinement | 20267 (1009) | 14311 (1423) | 45567 (4538) |
| Reflections used for R-free | 2000 (100) | 741 (78) | 1174 (123) |
| R-work | 0.2378 (0.4165) | 0.2424 | 24.20 (0.369) |
| R-free | 0.2739 (0.4274) | 0.2664 | 0.273 (0.3874) |
| Number of non-hydrogen atoms | 3782 | 3191 | 39474 |
| RMS(bonds) | 0.007 | 0.07 | 0.003 |
| RMS(angles) | 1.15 | 1.09 | 0.692 |
| Ramachandran favored (%) | 92.0 | 92.7 | 95.3 |
| Ramachandran allowed (%) | 7.3 | 6.8 | 4.1 |
| Ramachandran outliers (%) | 0.7 | 0.5 | 0.6 |
| Clash score | 1.1 | 4.7 | 4.5 |
| Average B-factor (Å^2^) | 84.6 | 200.0 | 395.3 |
