# Supplementary material for: Structural Basis for Properdin Oligomerization and Convertase Stimulation in the Human Complement System
Source: Front Immunol. 2019 Aug 22;10:2007. doi: 10.3389/fimmu.2019.02007 (PMC6713926; doi:10.3389/fimmu.2019.02007)
Supplement: Supplementary file 8 [file Table_2.DOCX]

**Supplementary table 2**. Data collection and refinement statistics for the structures where data collection statistics are not previously published. Statistics for the highest-resolution shell are shown in parentheses.

| **Structure** | **hFPNb1** | **hFPNb1- FPcΔ3- C3bBb-SCIN** | **C3-hC3Nb1** | **FPcΔ3 S-SAD*** |
| --- | --- | --- | --- | --- |
| PDB entry | 6RU3 | 6RUV | 6RU5 | 6RV6 |
| Wavelength (Å) | 0.9763 | 0.9762 | 0.9763 | 2.48 |
| Resolution range (Å) | 33.85-1.26  (1.305 - 1.26) | 49.37 - 6.2  (6.421 - 6.2) | 45.92 - 3.7  (3.832 - 3.7) | 69.5 - 3.508  (3.633 - 3.508) |
| Space group | R 3 2 :H | P 21 21 21 | C 1 2 1 | I 41 |
| Unit cell | 65.12 65.12 169.26 90 90 120 | 123.99 354.03 367.84 90 90 90 | 228.32 140.72 95.43 90 100.508 90 | 219.79 219.79 47.44 90 90 90 |
| Total reflections | 1330007 (63201) | 490733 (51314) | 179495 (5016) | 558237 (52726) |
| Unique reflections | 37557 (3561) | 37548 (3663) | 30601 (2522) | 27800 (2825) |
| Multiplicity | 35.4 (17.7) | 13.1 (14.0) | 5.9 (2.2) | 20.1 (18.7) |
| Completeness (%) | 99.33 (94.68) | 99.49 (99.92) | 97.32 (79.73) | 99.86 (98.91) |
| Mean I/sigma(I) | 34.26 (0.86) | 12.44 (0.59) | 6.10 (0.31) | 12.17 (1.15) |
| Wilson B-factor (Å^2^) | 22.52 | 435.84 | 141.98 | 134.82 |
| R-merge | 0.05304 (3.468) | 0.1609 (3.766) | 0.2188 (2.12) | 0.1888 (2.84) |
| CC_1/2_ | 1 (0.388) | 0.999 (0.344) | 0.995 (0.0977) | 1 (0.404) |
| Reflections for refinement | 37510 (3542) | 37478 (3661) | 30921 (2518) | 27800 (2824) |
| Reflections for R-free | 1886 (184) | 1937 (188) | 1885 (153) | 1354 (118) |
| R-work | 0.1866 (0.4253) | 0.2542 (0.4315) | 0.2743 (0.4051) | 0.2230 (0.3936) |
| R-free | 0.2017 (0.4223) | 0.2797 (0.4118) | 0.2879 (0.3950) | 0.2721 (0.4616) |
| CC(work) | 0.957 (0.656) | 0.943 (0.474) | 0.949 (0.297) | 0.949 (0.571) |
| CC(free) | 0.956 (0.677) | 0.859 (0.411) | 0.924 (0.459) | 0.918 (0.580) |
| Non-hydrogen atoms | 1099 | 41916 | 13874 | 3191 |
| protein | 986 | 41278 | 13799 | 2958 |
| Protein residues | 124 | 5295 | 1749 | 406 |
| RMSD (bonds Å/angles °) | 0.011/1.15 | 0.005/0.98 | 0.003/0.68 | 0.005/0.60 |
| RamachandranFavored/allowed/outliers (%) | 98.4/1.6/0.0 | 95.20/4.0/0.8 | 92.6/7.3/0.1 | 93.7/5.3/1.0 |
| Rotamer outliers (%) | 0.00 | 2.64 | 0.48 | 4.31 |
| Clashscore | 0.00 | 2.29 | 5.57 | 5.04 |
| Average B-factor (Å^2^) | 31.72 | 527.86 | 180.79 | 153.03 |
