# Supplementary material for: Structural Basis for Properdin Oligomerization and Convertase Stimulation in the Human Complement System
Source: Front Immunol. 2019 Aug 22;10:2007. doi: 10.3389/fimmu.2019.02007 (PMC6713926; doi:10.3389/fimmu.2019.02007)
Supplement: Supplementary file 9 [file Table_3.DOCX]

**Supplementary table 3. FP residues close to C3b and Bb in the FP-convertase complex.** Residues are listed if they are within 3.8 Å of C3b or Bb or minor changes to their side chain conformation could result in a FP-C3b or FP-Bb contact.

| **FP residue** | **repeat** | **C3b residue** | **Bb residue** |
| --- | --- | --- | --- |
| W318 | TSR5 | Q1638 |  |
| R329 | TSR5 | F1659 | L349, K350 |
| R330 | TSR5 | S1655, F1659, P1662 |  |
| N331 | TSR5 | P1662 |  |
| M332 | TSR5 |  | K348 |
| E339 | TSR5 |  | L349, K348 |
| I340 | TSR5 |  | L349 |
| P341 | TSR5 | F1659 |  |
| Q343 | TSR5 | V1658 |  |
| C350 | TSR5 | Q1638 |  |
| R353 | TSR5 | Q1638, D1639 |  |
| R359 | TSR5 | E1634, D1635, Q1638 |  |
| C360 | TSR5 | E1634 |  |
| G362 | TSR5 | E1634 |  |
| Q364 | TSR5 | Q1638, Q1643, Q1647 |  |
| Q365 | TSR5 | Q1647 |  |
| I367 | TSR5 | A1651, E1654 |  |
| H369 | TSR5 | V1658 |  |
| M420 | TRS6 | V1658, F1659 | Y317, K350 |
| V421 | TRS6 | V1657, V1658 | Y317 |
| E422 | TSR6 |  | Y317 |
| K427 | TRS6 | V1658 |  |
